# The Effects of Compensatory Scanning Training on Mobility in Patients with Homonymous Visual Field Defects: A Randomized Controlled Trial

**DOI:** 10.1371/journal.pone.0134459

**Published:** 2015-08-14

**Authors:** Gera A. de Haan, Bart J. M. Melis-Dankers, Wiebo H. Brouwer, Oliver Tucha, Joost Heutink

**Affiliations:** 1 Department of Clinical and Developmental Neuropsychology, University of Groningen, Groningen, The Netherlands; 2 Royal Dutch Visio: Centre of Expertise for Blind and Partially Sighted People, Haren, The Netherlands; 3 Department of Neurology, University Medical Center Groningen, Groningen, The Netherlands; University of Regensburg, GERMANY

## Abstract

**Introduction:**

Homonymous visual field defects (HVFD) are a common consequence of postchiasmatic acquired brain injury and often lead to mobility-related difficulties. Different types of compensatory scanning training have been developed, aimed at decreasing consequences of the HVFD by changing visual scanning.

**Aim:**

The aim of the present study is to examine the effects of a compensatory scanning training program using horizontal scanning on mobility-related activities and participation in daily life.

**Method:**

The main interest of this study is to assess the effectiveness of training on mobility-related activities and participation in daily life. Visual scanning tests, such as dot counting and visual search, and control measures for visual functions and reading have been included as well. First, it is examined how performance on scanning and mobility-related measures is affected in patients with HVFD by comparing scores with scores of a healthy control group (n = 25). Second, the effect of training is assessed using an RCT design, in which performance of 26 patients before and after training is compared to performance of 23 patients in a waiting list control group.

**Results:**

Self-reported improvements after training were found, accompanied by improvements in detecting peripheral stimuli and avoiding obstacles during walking, especially in dual task situations in which a second task limits the attentional capacity available for compensatory scanning. Training only improved mobility-related activities in which detection of peripheral stimuli is important, while no improvement was found on tests that require other visual skills, such as reading, visual counting and visual search.

**Conclusion:**

This is the first RCT to evaluate the effects of a compensatory scanning training that is based on a systematic horizontal scanning rhythm. This training improved mobility-related activities. The results suggest that different types of compensatory scanning strategies are appropriate for different types of activities.

**Trial Registration:**

ISRCTN Registry ISRCTN16833414

## Introduction

Homonymous visual field defects (HFVDs) are a common consequence of acquired brain damage and refer to visual field defects similar for both eyes and contralateral to the brain damage. The most common form of a HVFD is homonymous hemianopia, in which the left or the right half of the visual field is not perceived. Homonymous hemianopia is estimated to occur in 8–31% of all stroke patients [1,2], but can also be caused by traumatic brain injury, brain tumor, or other pathologies (e.g. multiple sclerosis, epileptic disorders, MELAS, and the posterior form of Alzheimer disease)[3,4].

After one month, spontaneous recovery of the visual field, at least partly, is seen in 50% to 69% of patients with hemianopia [5–7]. Most patients become aware that they should compensate by looking towards the blind side. However, spontaneous recovery and spontaneous compensation are often insufficient so that considerable difficulties with activities in daily life and independent living remain [8]. Patients with HVFDs often report difficulty in scanning their surroundings fast enough to detect all objects and people in time, leading to feelings of insecurity and difficulty with orientation and mobility [9]. This is illustrated by the finding that in a group of patients with HVFD referred for low-vision rehabilitation, almost 90% indicated they frequently collide with people or objects on the side of the HVFD [10]. These mobility problems often restrict participation in society considerably and may lead to marked impairments of quality of life [8,11–13].

Compensatory scanning training (CST) aims to decrease the impact of the visual field defect by enlarging the functional field of view through optimizing visual scanning. Based on different rationales, several CST programs have been developed. Most programs include computerized exercises to stimulate compensatory scanning and these exercises can be divided in three categories. The first type of exercise is based on visual search in which patients have to find one or more targets among distractors [9,14–22]. Exercises of the second type focus on finding a target not surrounded by distractors, with the target appearing at unpredictable positions [23–30]. In the third type of exercise participants make fast and large saccades towards targets presented on the horizontal axis specifically [9,15,22,31,32]. Some CST programs combine these different types of exercises or apply additional exercises, such as copying complex drawings. Only a few CST programs include exercises to practice transfer of the adapted scanning behavior to activities of daily life [9,15,20,27,28,30,31].

Previous studies on the effect of CST have been encouraging, but the impact on activities of daily living is unclear [33–36]. In many studies part of the tests used to assess the effect of training tended to be very similar to the exercises practiced during training [9,14–19,21,23,24,26,31]. Very few studies incorporated mobility-related tests [14,30,31]. Only little evidence has been found for transfer of CST effects to activities of daily life beyond the specific tasks that were trained. Furthermore, the majority of these studies used within-subjects designs. A small number of the effect studies on training with visual search exercises used a randomized controlled trial (RCT) design to compare the effects of CST with a control group [14,16,19–21], but no RCTs have been performed for training with a focus on horizontal scanning strategies. In conclusion, a well-designed study on the effects of CST on mobility-related activities and participation is needed.

The aim of the present study is to examine the effects of a CST program on an extensive set of scanning and mobility-related measures. This CST teaches patients with HVFDs a systematic scanning rhythm using exercises of horizontal scanning. The main interest of this study was to examine the effects of training on mobility-related activities and participation. Visual scanning tests, such as dot counting and visual search, were included as well, in order to examine various underlying visual performance and in order to enable comparison with the previous studies on the effects of CST. Furthermore, control measures for visual functions and reading have been included. It was hypothesized that this CST would improve scanning and mobility related activities, while visual functions, such as visual field size, would not be affected by the intervention. No effect on reading was expected, since two previous studies found no effect of CST on reading performance [16,19]. While reading relies on small saccades, this training focusses on large horizontal saccades. First, it is examined how performance on scanning and mobility-related measures is affected in patients with HVFD by comparing these patients with a healthy control group. Second, the effect of training is assessed using an RCT design, in which performance of patients before and after training is compared to performance of patients in a waiting list control group.

## Materials and Methods

### Ethics

The protocol for this trial and supporting CONSORT checklist are available as supporting information; see S1 Checklist and S1 Protocol. The study protocol was approved by the Medical Research Ethics Committee of the University Medical Center Groningen (registration number METc 2010/078) and by the relevant patient organizations. This study was registered at the Central Committee on Research Involving Human Subjects (CCMO; www.ccmo.nl/en; registration number NL31718.042.10). The study was registered as a clinical trial at the ISRCTN Registry [ID ISRCTN16833414; URL http://www.isrctn.com/ISRCTN16833414]; Registration occurred after the trial began since the research group was not aware that this study design required public registration as a clinical trial. The authors confirm that all ongoing and related trials for this intervention are registered. The study was performed in accordance with the 2008 Declaration of Helsinki. All participants gave their informed written consent. For all participants, there was no reason to doubt their capacity to consent, since they all had the capacity to sign the rehabilitation contract themselves and to formulate their individual goals for rehabilitation during the registration stage at the rehabilitation center, and they all had MMSE scores ≥ 24 out of 30.

### Design

**Fig 1:**
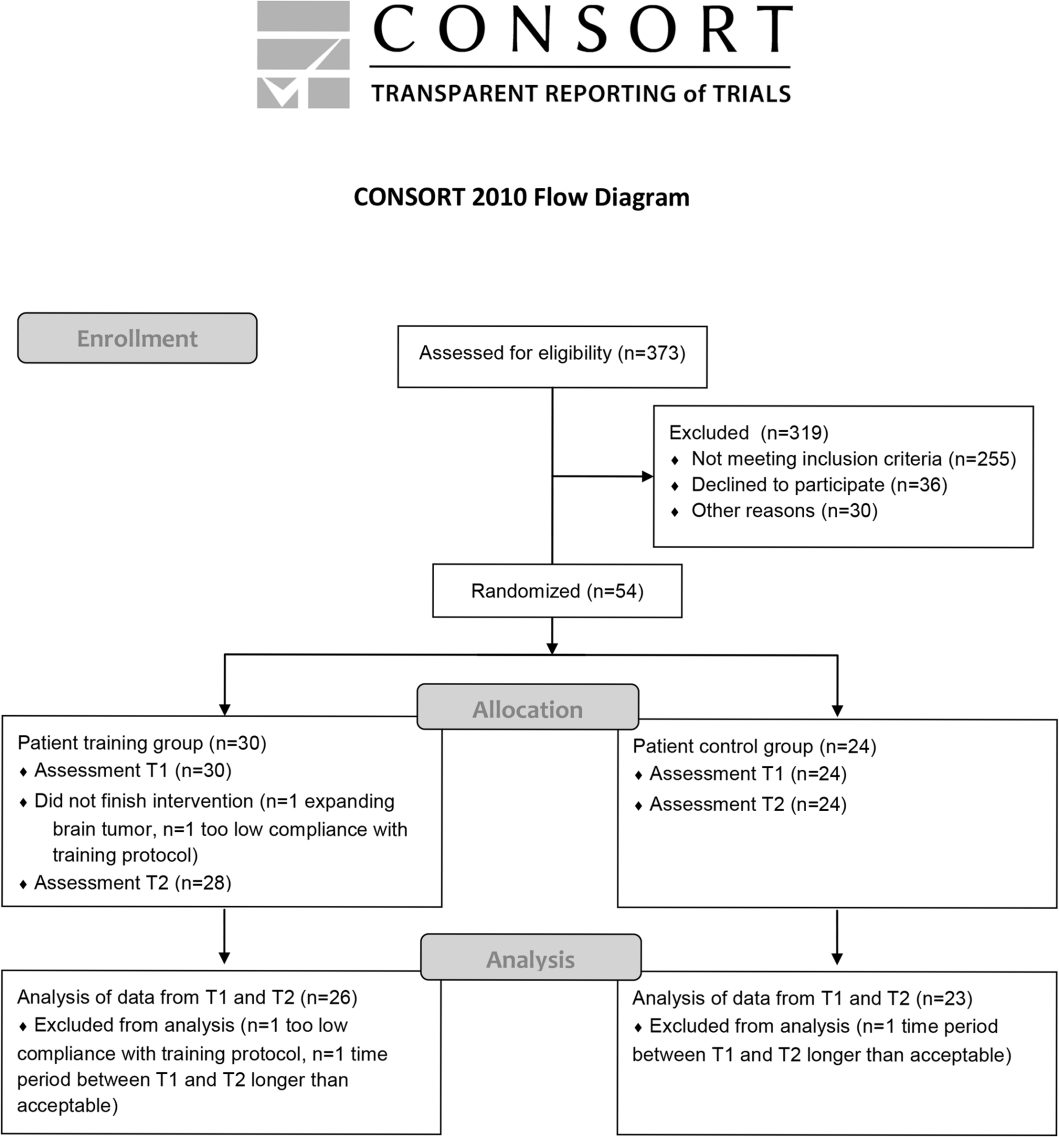
Consort statement flow chart.

Patients with HVFD were assigned to either the training group or the waiting list control group. The flow chart of the study is presented in Fig 1. Patients in the training group were assessed the week before training (T1) and the week after training (T2). Patients in the waiting list control group also participated in two assessments, but received no training in between. Time between assessments was 13 weeks for both groups. This was 16 weeks for one included patient of the training group, because he cancelled T2 because of other private and work-related engagements, and a new appointment could be made for three weeks later (there was no breach of training, no further training after 13 weeks and his scores were not outliers). The assessments and training took place between March 2010 and October 2012. For patients in the training group, training could be extended with a number of sessions after T2 outside the scope of this study, dependent on the mobility goals set out at the start of the training. Patients in the waiting list control group were offered training after T2.

Allocation to the groups occurred by the method of minimization [37]. This is a dynamic procedure that calculates for a new participant the difference for each of a set of predefined, dichotomous factors, based on the characteristics of the participants already included in the two groups. The method of minimization is demonstrated to be superior to complete randomization for the sample size in our study [38]. Differences between the groups were minimized regarding gender, side of field defect (left or right), size of field defect (hemianopia vs. quadrantanopia), age (younger vs. older than 55), and time since onset (shorter vs. longer than 12 months). Because time since onset was assumed less important than the other variables, this variable was weighted less heavily (0.5) than the others (1.0). Upon inclusion of a new participant, author GH entered the characteristics of the patient into the randomization software that contained the characteristics of the previously included patients, which resulted in allocation to the training group or the waiting list control group.

For the healthy control group, the scanning and mobility-related tests were administered at T1 only. Assessments of the healthy control participants took place between October and December 2012.

Performance of the two patient groups at T1 are compared to performance of the healthy control group and changes between T1 and T2 in the training group are compared to changes in the waiting list control group.

### Sample size

The sample size was based on previous studies on the effect of CST (in terms of reaction times, eye movement parameters, data from ADL tasks and questionnaire data)[18,22]. Taking the lowest value encountered (effect size = 0.65), a minimum of 30 participants per group would be required (training group vs. waiting list control group; two independent groups; α = 0.05; β = 0.20; one-sided testing). When comparing pre and post assessments within a group of 60 participants, effect sizes of 0.34 can be detected with power 0.80 and one-sided testing with 0.05 significance. This means that even in case of low effectiveness of compensatory scanning training, a group size of n = 60 would be fully sufficient. Therefore, the aim was to recruit 30 participants for each patient group.

### Participant recruitment

Patients were recruited at Royal Dutch Visio and Bartiméus, the two centers of expertise for blind and partially sighted people in the Netherlands. The main inclusion criterion was presence of a HVFD, at least a quadrantanopia, restricted to one half of the visual field, due to acquired postchiasmatic brain injury. Visual field defects that covered the major parts of two quadrants were regarded as hemianopia, while smaller field defects were classified as quadrantanopia. In order to minimize the chance of spontaneous visual field recovery, time since onset had to exceed 5 months, minimizing chances for spontaneous recovery of the field defect. Between January 2010 and July 2012, 373 patients suspected of having such an HVFD were registered. In order to examine the inclusion criteria, patients underwent extensive and standardized ophthalmological and neuropsychological assessments at the centers mentioned above prior to participation in the study. The following tests were included in the neuropsychological assessments: Mini Mental State Examination, Eight word test, Hospital Anxiety and Depression Scale, Trail Making Test, Visual Object and Space Perception, Balloons, Drawings, Line Bisection, Rey Complex Figure Test, and behavioral tests for optic ataxia and sticky fixation. To be included, patients required a minimum binocular visual acuity of Snellen 0.5 (6/12 or 20/40, LogMAR 0.3), a stable neurological and ophthalmological condition, non-disturbed eye and head motility, ability to walk at least 50 meters, and a MMSE score ≥ 24 out of 30. Exclusion criteria were ocular diseases affecting the visual field or binocular visual acuity, signs of severe physical impairments or (neuro)psychological disorders. Neglect was excluded based on the Balloons, drawings, Line Bisection and Rey Complex Figure Test.

Besides patients with HVFD, healthy control participants without visual disorders and without brain damage were recruited. They were only included in the study when they were confirmed not to have physical, neurological or psychological impairments that constrain mobility. Binocular visual acuity had to exceed Snellen 0.8 (6/7.5 or 20/25, LogMAR 0.1) and MMSE scores of at least 24 out of 30 were required. The healthy control group was matched with the patient group regarding age and level of education. Recruitment of healthy control participants took place in October and November 2012.

### Training

The Template for Intervention Description and Replication checklist is available as supporting information; see S2 Checklist. The training protocol was developed at Royal Dutch Visio and abbreviated as IH-CST (InSight-Hemianopia Compensatory Scanning Training). Training according to this protocol was provided in Dutch at nine locations of Royal Dutch Visio and one location of Bartiméus in the Netherlands. Training was given by occupational therapists that followed complementary theoretical and practical in-service education on the IH-CST protocol. They were extensively supervised by two therapists with years of experience with the training paradigm. The training consisted of 15 individual sessions of 60–90 minutes each, 18.5 hours of face-to-face training in total during a period of 10 weeks. The aim of the IH-CST is to teach patients with HVFD to apply a systematic, anticipatory scanning strategy in order to compensate for their visual field defect during a wide range of mobility-related activities. Similar to the training described by Tant [31], patients are taught a scanning strategy consisting of a triad of horizontal saccades. In the IH-CST, the scanning strategy is to start with one large saccade towards the blind side, followed by a large saccade ending on the peri-central seeing side, and then back to the starting point of looking straight forward. The large saccade from the center towards the blind side is 44 degrees of visual angle at maximum. This is the largest saccade most people can make without moving the head. Patients learn to generate this scanning rhythm endogenously on an anticipatory basis and to adjust the speed of repetition of this scanning rhythm to environmental demands and to the speed of walking, cycling, etc. The underlying idea is that early detection of obstacles is of high importance during mobility. When an obstacle is detected, one can anticipate to the situation in order to avoid collision with the obstacle. For patients with HVFD, the reduced visual input makes it challenging to create and sustain a proper visual overview. In order to compensate for the loss of visual information caused by the visual field defect, frequent application of large saccades towards the blind side is needed.

**Fig 2:**
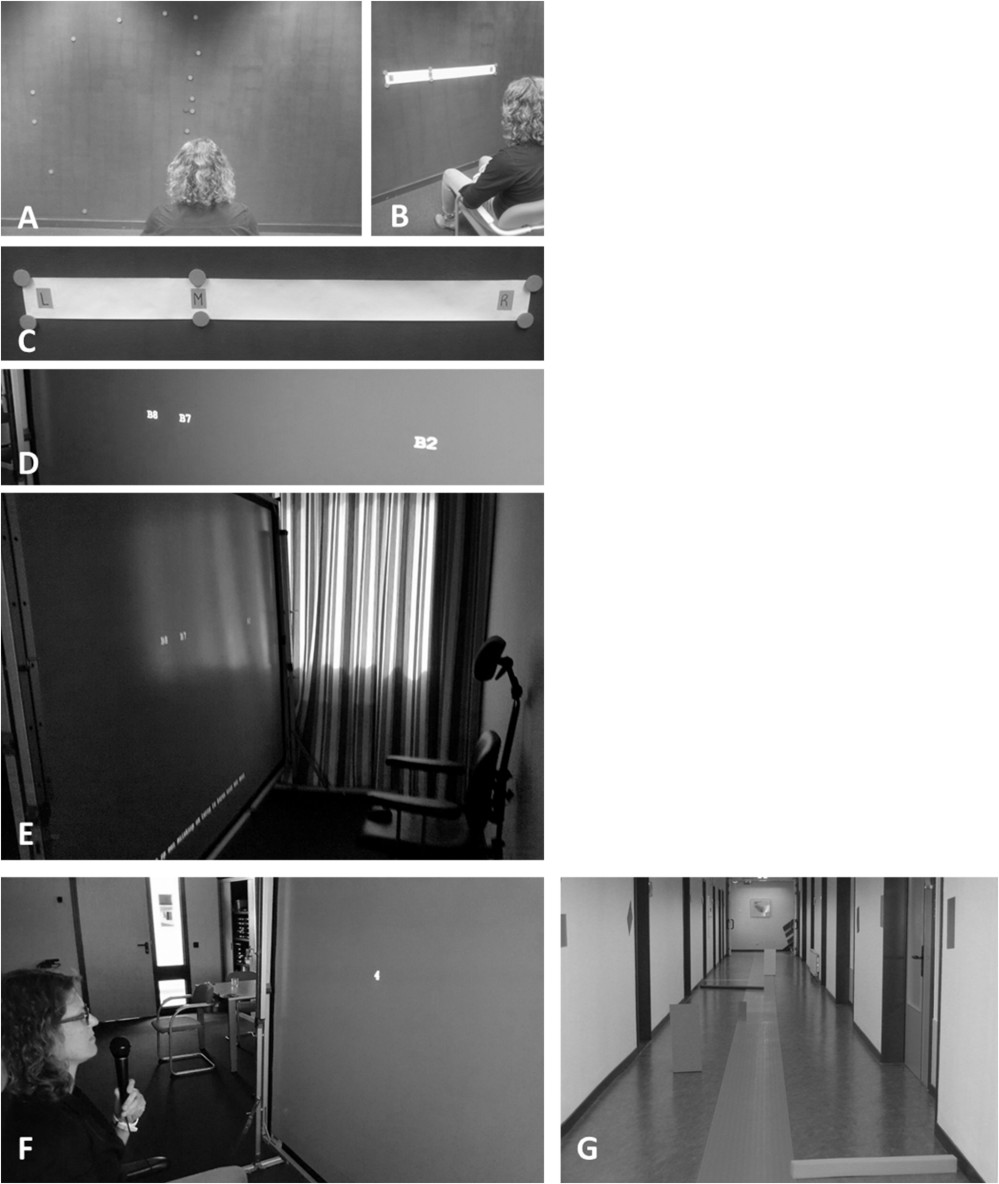
Pictures illustrating different elements of the IH-CST, example for right-sided hemianopia. (A) Example of exercises aimed at improving awareness of the size and shape of the visual field defect. The patient is asked to focus at a target in front and indicate the borders of the visual field. Accordingly, the visual field is plotted on the wall with stickers or magnets. (B) Pieces of paper with letters M (middle), R (right) and L (left) used to practice the scanning rhythm. First the paper is laying on a table, then it is attached to a wall in front of the patient (C). The same scanning triad is then presented on a large screen (D). The patient sits in front of this screen in a chair with a head rest (E). Numbers are presented one by one in the order of the scanning triad. The patient has to read the numbers out loud and a microphone is used to record responses (F). After each exercise, the reaction times for targets left, middle and right are presented on the screen. The scanning rhythm is systematically practiced with several exercises gradually increasing speed and amplitude of the scanning triad. (G) A corridor filled with obstacles to practice use of the scanning rhythm during walking. This will be succeeded by practice in a range of daily life mobility situations, with increasing complexity and cognitive load, such as walking in busy shopping areas.

Fig 2 illustrates different elements of the IH-CST. The protocol starts with exercises for improving awareness of the size and shape of the visual field defect and its consequences for daily life activities. Then, the scanning rhythm is systematically practiced with several exercises gradually increasing speed and amplitude of the scanning triad. At first, only eye movements are allowed, since eye movements are faster than head movements, they do not lead to neck-muscle complaints and they naturally precede head movements. In a later stage, head movements following the eye movements are practiced to increase the range of scanning. A substantial part of the training is dedicated to the practicing of the scanning rhythm in a range of daily life mobility situations, with increasing complexity and cognitive load, in order to optimize transfer to visual activities and participation in daily life. For every exercise, specific targets are defined for speed and amplitude of the scanning rhythm as well as transfer to an activity of daily life, which must be reached before the participant proceeds to the next exercise. For example, practicing the scanning rhythm during cycling will only be started after scanning during walking is performed without problems. Depending on the needs of the patient, some other compensatory techniques are practiced, for example searching for an object on a shelf. The main reason for including these exercises is that they are expected to increase insight into the field defect. The focus of the IH-CST, however, is on applying a systematic, anticipatory scanning rhythm during a wide range of mobility-related activities.

#### Homework assignments

In order to stimulate transfer to daily life, homework assignments are included in the training protocol. The first homework assignment is aimed at improving insight in the visual field defect. In this assignment, the patient has to answer a number of questions on what they see and cannot see when looking straight forward in a number of predefined situations.

Further homework assignments are aimed at practicing the scanning rhythm in daily life situations, stimulating transfer to daily life. Homework starts with practicing the scanning rhythm using pieces of paper (Fig 2C) three times a day for five minutes. First a smaller band is used and then a wider band, in order to increase the amplitude of the saccades.

When the patient is able to perform the scanning rhythm in the right way, the patient is encouraged to practice the scanning rhythm every day while moving around in different situations (no equipment used). The mobility situations build up from quiet, structured and familiar surroundings to busier and more complex and unfamiliar surroundings, depending on the progress the patient has made in applying the scanning rhythm. The homework instructions of the therapist are fitted for the individual patient, depending on the specific goals that were set by the patient at onset of the training. If these goals include cycling for example, then the homework assignments will also include practicing the scanning rhythm while cycling, again from practice in quiet surroundings to more complex surroundings.

Patients are asked to keep a diary of their practice at home and the therapists asks about the progress of the homework assignment at the beginning of every training session. These structured homework assignments are on the one hand aimed at encouraging practice in daily life, but on the other hand prevent the patient from practicing too difficult situations at an early stage of training. The ultimate goal of the training is that use of the scanning rhythm becomes an automated activity, naturally embedded in every mobility situation encountered in daily living.

### Assessments

#### Procedure

The assessments were performed by the department of Clinical and Developmental Neuropsychology of the University of Groningen and took place in the University Medical Center Groningen, the Netherlands. Participants were tested individually by assessors who were blinded to participants’ group allocation. Communication language during the assessment was Dutch. The results were anonymized and had no influence on training and rehabilitation at the rehabilitation center; no feedback on the results from the assessments was provided to Royal Dutch Visio or Bartiméus for individual patients. In order to increase insight in the degree of difficulty caused by the HVFD, the tests related to scanning and mobility were administered in a healthy control group as well, using the same setup and instructions as for the patient groups.

#### Tests for visual functions

Monocular visual acuity was tested using the ETDRS 2000 Letter Chart at 4 meters and 500 lux [39]. Contrast sensitivity was measured using the Gecko Test at 3 meters and 500 lux [40]. Monocular visual fields were plotted with Goldmann perimetry (isopters V-4, III-4 and I-4) while continuously checking stability of fixation. An independent orthoptist experienced with interpreting perimetry plots of HVFD patients, further analyzed perimetry output. Plots were recoded so that the orthoptist was unaware at which assessment the plot was made. Functional Field Score (FFS) [41,42] was calculated from the plots of isopter III-4 using the overlay grid from Langelaan [41], in which the center and lower half of the visual field weigh more heavily since they are deemed functionally more important. To check whether visual fields changed between T1 and T2, it was evaluated for every participant and each eye whether the border between the blind and intact part of the visual field had shifted at least 5°. For the healthy control group, it was only checked whether visual acuity exceeded Snellen 0.8 (6/7.5 or 20/25, LogMAR 0.1) and no other assessments were performed regarding visual functions.

#### Reading tests

Two different reading tests were administered. The Radner reading chart [43,44] consists of sentences with decreasing text size that have to be read out loud. Viewing distance was 40 cm. Outcome measures were average reading speed in sentences 3–7, as these sentences could be read by all participants, and minimal readable text size expressed in LogRad units. In a second reading test, participants read out loud a text of approximately 400 words. Participants were allowed to choose their preferred viewing distance while reading the text. After reading the text, participants answered two questions about its content. Reading speed and correct answers were measured. For both tests, preferred glasses or lenses were allowed. Three parallel versions of both reading tests were used in a Latin Square design (on T1 and T2 respectively, subject 1 completed versions 1 and 2, subject 2 completed versions 2 and 3, subject 3 completed versions 3 and 1, etc.).

#### Basic scanning tests

**Fig 3:**
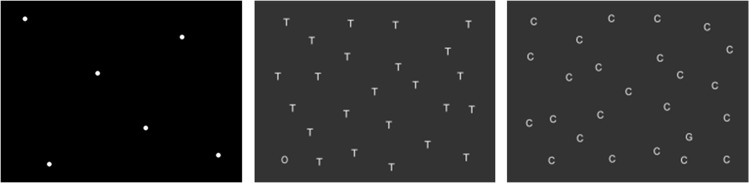
Examples of displays from the dot counting test, parallel search test and serial search test.

Three basic scanning tests were administered (Fig 3). In the first test, participants counted dots in 32 different dot patterns. Half of the trials contained few dots (6, 7, 8 or 9 dots, 4 trials each); the other half contained many dots (18, 19, 20 or 21 dots, 4 trials each). Order of trials was randomized once and the same order was applied to all participants at all assessments. The second test was a visual search test in which participants indicated whether or not the letter O was present among T’s (parallel search), while in the third test presence of the letter G among C’s was questioned (serial search). Stimuli were presented on a large screen (40° horizontally and 33° vertically) with a viewing distance of 192 cm. Participants were allowed to move their head while scanning. No instructions on how to scan the images were given. Reaction times as well as accuracy scores were recorded. For the dot counting test, reaction times and proportion of correct responses were also calculated for trials with few dots and trials with many dots separately. For the visual search tests, reaction times were analyzed by target trials and non-target trials as well. Besides the total number of errors, i.e. omission errors plus commission errors, the number of omission errors was analyzed separately.

#### Hazard perception test

The hazard perception test is described in more detail by Vlakveld [45]. Twenty-five photos of traffic situations were presented from the view point of a car driver. After looking at each photo for eight seconds, participants choose whether in the given situation they would brake, release the accelerator or keep the same speed (i.e. no intervention). In the current study, size of the photos was 40° by 25° and viewing distance was 192 cm. Head movements were allowed and no instructions on scanning strategies were provided. Besides the number of incorrect responses (absolute error rate), the adapted error rate and risk-index were calculated. The adapted error rate was calculated by the amount of incorrect responses, with very risky responses (“no intervention” when the correct response is “braking”) and very cautious responses (‘braking” when the correct response is “no intervention”) counting as two errors. The risk-index was defined by the proportion of risky answers (risk-index = (2*very cautious responses + cautious responses) / adapted error rate).

#### Tracking Task

The Tracking Task is a test of divided attention based on an earlier version described by Brouwer [46]. Participants were seated in front of a simple driving simulator, in which they were driving on a straight road with fixed speed. Participants first practiced use of the steering wheel for one minute. They were then instructed to maintain a stable position on the middle of the right lane. This required continuous attention because of an imaginary cross-wind influencing the lateral position on the road. During a three-minute cross-wind assessment, maximum cross-wind was determined for which deviation in lateral position was still within predefined limits, followed by a two-minute practice of driving with this amount of cross-wind. Two peripheral screens on which arrows were presented, were positioned on the left and right of the driving simulator. One arrow at a time was presented and the locations (left or right screen) proceeded in a non-predictive order. Participants pressed the button on the steering wheel corresponding to the pointing direction of the arrow (i.e. left or right) as fast and accurate as possible. In case the participants did not respond within 5 seconds, the arrow disappeared and no reaction time was registered. In the single task condition, no steering was required because position on the road was fixed. This condition continued for two minutes, preceded by one minute of practice. During the dual task condition, lane tracking and peripheral detection were combined. This condition lasted for six minutes, preceded by two minutes of practice. The cross-wind strength as individually determined during the cross-wind assessment of T1 was applied in the dual task conditions of both T1 and T2.

Head movements were allowed since these are part of natural scanning behavior. Standard deviation in lateral position on the road (SDLP), as well as omission errors, number of faulty responses and reaction times for the peripheral stimuli were recorded for the dual task condition. The dual-to-single-task-ratio (DSR) was calculated by dividing the mean reaction time in the dual task condition by the mean reaction time in the single task condition.

#### Obstacle course

The effects of obstacles and cognitive load on walking speed were examined in a standardized obstacle course inside the hospital. Participants were asked to walk through a straight corridor with a comfortable pace, turn around at the end and walk back. Total length (back and forth) of the course was 35 meters. First, the corridor was free of obstacles and preferred walking speed was measured. Then participants walked through the empty corridor while cognitive load was added by asking the participant to repeat verbally presented digit series while walking. Length of the digit series was equal for T1 and T2 and matched the maximum amount of digits the participant was able to repeat correctly as determined beforehand (with the WAIS-Digit Span Forward). Subsequently, participants walked through the corridor filled with 32 obstacles. These were obstacles that could be encountered in real life, such as chairs and litter bins. The obstacles were positioned in a standardized way and participants had to sway through the course in order to avoid touching the obstacles. The obstacles course was first walked with and then without the cognitive dual task.

Contact with obstacles and proportion correct answers on the digit series (Digit Score) were analyzed for the condition with obstacles and with cognitive load. The percentage preferred walking speed (PPWS) was calculated by dividing the walking speed in the obstacle course with cognitive load by the walking speed in the obstacle free corridor with cognitive load.

#### Questionnaires

Three standardized questionnaires were applied to assess the impact of the HVFD on activities and participation in daily life. In the Visual Functioning Questionnaire (NEI-VFQ-25) [47,48], participants rate the impact of their visual impairment on several health-related domains, such as emotional well-being, social functioning and a number of activities. The Independent Mobility Questionnaire (IMQ) [49] assesses the level of difficulty the participant experiences because of visual impairment in a wide range of mobility-related situations. The Cerebral Visual Disorders questionnaire (CVD) consists of two parts. The first part was originally developed by Kerkhoff and colleagues [50] and asks the participant whether nine vision-related problems were experienced or not. The second part consists of questions about the level of difficulty experienced in twelve specific activities (Dittrich, 1996, as cited in [31], p.75). The questionnaires were administered during a structured interview, i.e. orally, since reading difficulties are common in patients with HVFD. The three total scores of the questionnaires yielded the main outcome measures. For the NEI-VFQ-25, higher scores indicate less difficulty experienced by the patient, while for the IMQ and CVD, higher scores refer to more difficulty.

### Statistical Analysis

Participant characteristics were compared between the patient training group (P-TRAINING), the patient waiting list control group (P-WAITING) and the healthy control group (HEALTHY) using ANOVA and post-hoc tests (Least Significant Difference) for age and level of education, two-tailed independent samples t-Test for FFS and time since onset, and two-tailed Chi-Square Test for gender, etiology and side of HVFD. Test performance in the two patient groups at T1 was compared to test performance of the healthy control group with a two-tailed independent samples t-Test. The effect of training was examined by the group*time interaction effects from General Linear Model (GLM) Repeated Measures analysis, with group (P-TRAINING vs. P-WAITING) as the between-subjects factor and time (T1 vs. T2) as the within-subjects factor. In the GLM, FFS at T1 was inserted as a covariate (except for the analyses on visual functions), because FFS at T1 was significantly higher for the waiting list control group than for the training group (t(38.2) = -2.08, *P* = 0.045). Within-group changes between T1 and T2 were examined with two-tailed matched pairs t-Test for the training group and waiting group separately. In case of a significant interaction effect in the GLM, the two patient groups were compared using a two-tailed independent samples t-Test for T1 and T2 separately. Changes in the border of the visual field defects were analyzed with a Chi-square test, comparing the distributions between the training group and waiting list control group.

There was no evidence for serious violations of the assumptions for all statistical tests. For the two-tailed independent samples t-Test, the assumption of equal variances in the two groups was tested with Levene’s test for equality of variances. In case equal variances cannot be assumed, the unequal-variance t-Test was performed. Cases with missing values (because of measurement flaws, technical bugs or shortage of testing time e.g. in case of late arrival due to rush hour) were excluded pairwise. Significant effects were defined by *P*-values < 0.050. In case of a *P*-value below 0.100, the exact *P*-value is reported. Effect sizes belonging to the group*time interactions were calculated with the formula for effect size estimate d_ppc2_ as described by Morris [51]. Effect sizes for the within-group and between-group comparisons were calculated according to Cohen’s d [52]. Effect sizes were classified as negligible (d < 0.20), small (d > 0.20), medium (d > 0.50) or large (d > .80).

## Results

The individual-level data are provided in S1 File.

### Participants

Fifty-four patients with unilateral HVFD were included and data from 49 patients were analyzed. Forty-eight patients received training at Royal Dutch Visio and one at Bartiméus. According to the procedure of minimization [37], 26 patients were allocated to the training group and 23 to the waiting list control group. Twenty-five healthy control participants were included. The healthy control group contained less men than the combined patient groups (χ(1) = 9.24, *P* = 0.002). Participants’ characteristics are summarized in Table 1. No important harms caused by the training or the assessments were encountered, nor reported by the participants.

**Table 1:** Summary of participant characteristics (mean ± SD, range).

		Training group (n = 26)	Waiting list control group (n = 23)	Healthy control group (n = 25)	*P*-value
Gender	Men	18	14	7	0.002^b^ (Chi^2^ Test, combined patient group vs. HEALTHY);0.539 (Chi^2^ Test, P-TRAINING vs. P-WAITING)
	Women	8	9	18	
Age (years)		55 ± 10.1 [27;70]	57 ± 13.0 [29;74]	53 ± 14.5 [28;76]	0.639 (ANOVA); 0.732 (post-hoc P-TRAINING vs. HEALTHY); 0.351 (post-hoc P-WAITING vs. HEALTHY)
Level of education ^a^		5.3 ± 0.8 [4;7]	5.3 ± 1.1 [2;7]	5.5 ± 0.8 [4;7]	0.624 (ANOVA); 0.399 (post-hoc P-TRAINING vs. HEALTHY); 0.406 (post-hoc P-WAITING vs. HEALTHY)
Etiology	iCVA	18	18		0.953 (Chi^2^ Test)
	hCVA	3	2		
	TBI	2	1		
	PHT	1	0		
	AVM extirpation	0	1		
	combined	2	1		
Side of HVFD	Left HVFD	18	15		0.765 (Chi^2^ Test)
	Right HVFD	8	8		
Visual field size	Functional Field Score (FFS)	58 ± 7.8 [48;80]	64 ± 11.4 [48;84]		0.045^b^ (t-Test)
	Quadrantanopia	5 (3 LL, 1 UL, 1 LR)	5 (3 LL, 2 UL)		
	Hemianopia	21	18		
Time since onset of HVFD (months)		18 ± 22.5 [5–122]	22 ± 24.6 [7;106]		0.528 (t-Test)

^a^ Level of education according to Verhage [53]; higher values represent higher levels of education.

^b^Significant difference (*P*-value < 0.050). iCVA = ischemic cerebrovascular accident, hCVA = hemorrhagic cerebrovascular accident, TBI = traumatic brain injury, PHT = penetrating head trauma, combined = combined etiology. LL = lower left quadrantanopia, UL = upper left quadrantanopia, LR = lower right quadrantanopia.

### Comparisons between healthy control participants and patients at T1

The mean test scores and standard errors of the means (SD) are presented in Table 2. The effect sizes of the comparisons are presented in Table 3. These tables also include the abbreviations of the parameters as referred to throughout the results section.

**Table 2:** Test scores (mean ± SD).

	Training group			Waiting list control group			Healthy control group	
	n	T1	T2	n	T1	T2	n	T1
		(Before training)	(After training)		(Early pre-assessment)	(Before training)		
**Tests for visual functions**								
Visual acuity right eye (VOD)	26	0.90 ±0.28	0.91 ±0.29	23	0.90 ±0.25	**0.99 ±0.26** b	-	
Visual acuity left eye (VOS)	26	0.97 ±0.26	0.97 ±0.25	23	0.90 ±0.26	0.92 ±0.26	-	
Contrast sensitivity	26	2.08 ±0.21	2.13 ±0.12	23	2.07 ±0.17	2.04 ±0.27	-	
Functional Field Score (FFS)	26	**57.92 ±7.80** c	57.75 ±6.74	23	63.79 ±11.41	62.58 ±11.13	-	
**Reading tests**								
Radner average reading speed(wpm)	24	153±31	159±33	21	146±41	147±34	-	-
Minimal readable text size (LogRad)	24	0.08 ±0.15	0.09 ±0.12	21	0.07 ±0.10	0.07 ±0.12	-	-
Text reading speed (wpm)	23	133±23	136±27	21	125±31	135±35	-	-
Text correct answers	24	1.46 ±0.66	**1.79 ±0.42** b	21	1.62 ±0.59	1.67 ±0.48	-	-
**Basic scanning tests**								
*Dot counting test*								
Reaction times (ms)								
All trials (Dots-RT-all)	21	**8904 ±3434** a	8515 ±3812	23	**9293 ±5142** a	8224 ±3201	25	6631 ±1496
Few dots (Dots-RT-few)	21	**5272 ±2315** a	4834 ±2012	23	**5115 ±1923** a	**4542 ±1408** b	25	3214 ±818
Many dots (Dots-RT-many)	21	**12495 ±4874** a	12207 ±6175	23	13471 ±8993	11942 ±5376	25	10048 ±2347
Proportion correct answers								
All trials (Dots-correct-all)	21	**0.72±0.17** a	0.75 ±0.18	23	**0.73 ±0.24** a	0.70 ±0.28	25	0.84 ±0.12
Few dots (Dots-correct-few)	21	0.93 ±0.09	0.91 ±0.20	23	0.88 ±0.22	0.85 ±0.28	25	0.96 ±0.07
Many dots (Dots-correct-many)	21	**0.50 ±0.29** a	0.59 ±0.29	23	0.57 ±0.31	0.56 ±0.34	25	0.72 ±0.21
*Parallel search test*								
Reaction times (ms)								
All trials (Par-RT-all)	21	**2369 ±785** a	2224 ±838	23	**2183 ±516** a	2140 ±545	25	1196 ±367
Target present (Par-RT-target)	21	**1539 ±562** a	1422 ±425	23	**1498 ±425** a	1416 ±389	25	996 ±260
Target absent (Par-RT-notarget)	21	**3199 ±1027** a	3027 ±1286	23	**2868 ±768** a	2861 ±793	25	1396 ±504
Accuracy								
Total number of errors (Par-err)	20	0.45 ±0.83	0.40 ±0.50	23	1.35 ±2.27	0.83 ±1.56	25	0.48 ±0.77
Number of omissions (Par-omis)	20	0.20 ±0.41	0.25 ±0.44	23	1.09 ±2.04	0.70 ±1.55	25	0.32 ±0.63
*Serial search test*								
Reaction times (ms)								
All trials (Ser-RT-all)	21	**5563 ±1592** a	5258 ±1541	23	**4998 ±2039** a	5196 ±2269	25	3498 ±1337
Target present (Ser-RT-target)	21	**3855 ±1472** a	3607 ±1031	23	**3602 ±1709** a	3676 ±1725	25	2600 ±1321
Target absent (Ser-RT-notarget)	21	**7270±1891** a	6909 ±2244	23	**6394 ±2607** a	6715 ±3123	25	4395 ±1474
Accuracy								
Total number of errors (Ser-err)	21	1.14 ±1.59	1.57 ±1.94	23	1.78 ±2.32	1.78 ±1.93	25	0.84 ±1.38
Number of omissions (Ser-omis)	21	0.95 ±1.12	1.48 ±1.81	23	1.61±2.04	1.74±1.94	25	0.76 ±1.39
**Hazard perception test**								
Absolute error rate	17	**10.41 ±2.43** a	9.24 ±2.33	22	9.32 ±2.77	8.68 ±3.00	24	8.50 ±2.21
Adapted error rate	17	**11.65 ±3.16** a	10.12 ±2.69	22	10.27 ±3.34	9.55 ±3.35	24	9.21 ±2.89
Risk-index	17	0.73 ±0.16	0.74 ±0.13	22	0.69 ±0.21	0.74 ±0.20	24	0.76 ±0.18
**Tracking Task**								
*Dual task condition*								
Reaction times (ms)								
All stimuli (TT-RT-all)	23	**1200 ±195** a	**1109 ±155** b	21	**1293 ±286** a	1271 ±341	25	943 ±147
Stimuli blind side (TT-RT-blind)	23	1487 ±267	1345 ±239	21	1539 ±451	1605±679	-	-
Stimuli seeing side (TT-RT-seeing)	23	**1014 ±146** c	982 ±165	21	1155 ±271	**1093 ±248** b	-	-
Stimuli blind side-stimuli seeing side	23	473 ±263	362 ±246	21	384 ±313	**512 ±514** e	-	-
Accuracy								
Number of faulty responses (TT-err)	24	0.67 ±0.92	0.92 ±1.02	21	1.19 ±1.12	0.76±1.14	25	0.84 ±1.07
Number of omissions (TT-omis)	24	0.29 ±1.00	0.08 ±0.28	21	0.38 ±1.12	0.57±1.54	25	0.04 ±0.20
Standard Deviation of Lateral Position (SDLP)	23	48.09 ±9.94	50.30 ±10.27	21	50.19±10.55	47.31 ±10.59	25	46.14 ±6.50
*Mean reaction time dual task divided by mean reaction time single task (dual-to-single-task-ratio*, *DSR)*								
All stimuli (DSR-all)	23	**1.27 ±0.22** a	**1.10 ±0.19** b	20	**1.17 ±0.20** a	**1.23 ±0.22** e	25	1.00 ±0.11
Stimuli blind side (DSR- blind)	23	**1.54 ±0.43** c	**1.29 ±0.38** b	20	1.29 ±0.31	**1.53 ±0.63** e	-	-
Stimuli seeing side (DSR- seeing)	23	1.07 ±0.20	1.06 ±0.19	20	1.07 ±0.22	1.06 ±0.18	-	-
**Obstacle course**								
Digit Score	24	**0.60 ±0.31** a	0.66 ±0.31	23	0.70 ±0.27	0.65 ±0.29	25	0.79 ±0.24
Number of contacts	24	**2.00 ±1.98** a	**0.88 ±0.90** b d	23	**2.52 ±2.59** a	1.74 ±1.60	25	0.48 ±0.65
PPWS	24	**46.36 ±9.75** a	**49.68 ±8.35** b	23	**48.33 ±8.67** a	49.72 ±11.02	25	57.98 ±7.88
**Questionnaires**								
NEI-VFQ-25 total score	26	66.30±12.56	**71.98±10.07** b d	23	64.10 ±14.30	**62.39±15.06** e	-	-
IMQ total score	26	2.48 ±0.70	**2.04 ±0.56** b d	23	2.57 ±0.68	**2.51 ±0.72** e	-	-
CVD total score	26	0.44 ±0.16	**0.36 ±0.13** b d	23	0.45 ±0.15	**0.46 ±0.16** e	-	-

^a^significant difference between the patient group and healthy control group at T1 (independent samples t-test, two-sided *P*-value < 0.050).

^b^significant within-group difference between T1 and T2 (matched pairs t-test, two-sided *P*-value < 0.050).

^c^significant difference between training group and waiting list control group at T1 (independent samples t-test, two-sided *P*-value < 0.050).

^d^significant difference between training group and waiting list control group at T2 (independent samples t-test, two-sided *P*-value < 0.050).

^e^significant Group (training vs. waiting list control) * Time (T1 vs. T2) interaction effect (GLM Repeated Measures, *P*-value < 0.050).

**Table 3:** Effect sizes for within-group and between-group comparisons (Cohen’s d [52]) and group*time interactions (d_ppc2_ as described by Morris [51]). Medium (d > 0.50) or large (d > 0.80) effects printed bold.

	Training vs. healthy at T1	Waiting list vs. healthy at T1	T1 vs. T2 for Training group	T1 vs. T2 for Waiting list group	Training vs. Waiting list at T1	Training vs. Waiting list at T2	Time*group Interaction
**Tests for visual functions**							
Visual acuity right eye (VOD)	-	-	0.08	0.45	0.01	0.27	0.29
Visual acuity left eye (VOS)	-	-	0.02	0.09	0.27	0.19	0.08
Contrast sensitivity	-	-	0.24	0.22	0.06	0.47	0.42
Functional Field Score (FFS)	-	-	0.04	0.24	0.61	**0.53**	0.11
**Reading tests**							
Radner average reading speed (wpm)	-	-	0.23	0.03	0.20	0.36	0.13
Minimal readable text size (LogRad)	-	-	0.09	0.00	0.09	0.18	0.06
Text reading speed (wpm)	-	-	0.13	0.43	0.32	0.03	0.27
Text correct answers	-	-	0.44	0.06	0.26	0.27	0.44
**Basic scanning tests**							
*Dot counting task*							
Reaction times (ms)							
All trials (Dots-RT-all)	**0.89**	**0.72**	0.13	0.29	0.09	0.08	0.15
Few dots (Dots-RT-few)	**1.23**	**1.31**	0.28	0.49	0.07	0.17	0.06
Many dots (Dots-RT-many)	**0.66**	**0.53**	0.06	0.23	0.13	0.05	0.17
Proportion correct answers							
All trials (Dots-correct-all)	**0.87**	**0.62**	0.23	0.18	0.05	0.17	0.24
Few dots (Dots-correct-few)	**0.41**	**0.52**	0.10	0.20	0.30	0.23	0.04
Many dots (Dots-correct-many)	**0.88**	**0.58**	0.39	0.07	0.23	0.09	0.32
*Parallel search task*							
Reaction times (ms)							
All trials (Par-RT-all)	**1.97**	**2.22**	0.14	0.19	0.28	0.12	0.15
Target present (Par-RT-target)	**1.28**	**1.44**	0.21	0.38	0.08	0.01	0.07
Target absent (Par-RT-notarget)	**2.29**	**2.29**	0.11	0.02	0.37	0.16	0.18
Accuracy							
Total number of errors (Par-err)	0.04	**0.52**	0.05	0.25	**0.51**	0.36	0.26
Number of omissions (Par-omis)	0.22	**0.52**	0.10	0.22	**0.58**	0.38	0.28
*Serial search task*							
Reaction times (ms)							
All trials (Ser-RT-all)	**1.42**	**0.88**	0.16	0.15	0.31	0.03	0.27
Target present (Ser-RT-target)	**0.90**	**0.66**	0.18	0.07	0.16	0.05	0.20
Target absent (Ser-RT-notarget)	**1.71**	**0.95**	0.14	0.18	0.38	0.07	0.29
Accuracy							
Total number of errors (Ser-err)	0.20	**0.50**	0.16	0.00	0.32	0.11	0.21
Number of omissions (Ser-omis)	0.15	0.49	0.24	0.08	0.40	0.14	0.24
**Hazard perception test**							
Absolute error rate	**0.83**	0.33	0.43	0.26	0.42	0.21	0.20
Adapted error rate	**0.81**	0.34	0.45	0.30	0.42	0.19	0.24
Risk-index	0.14	0.36	0.05	0.26	0.25	0.01	0.25
**Tracking Task**							
*Dual task condition*							
Reaction times (ms)							
All stimuli (TT-RT-all)	**1.49**	**1.58**	0.45	0.13	0.38	**0.62**	0.28
Stimuli blind side (TT-RT-blind)	-	-	0.42	0.16	0.14	**0.52**	**0.56**
Stimuli seeing side (TT-RT-seeing)	-	-	0.21	0.47	**0.66**	**0.53**	0.14
Stimuli blind side—stimuli seeing side	-	-	0.33	0.30	0.31	0.38	**0.82**
Accuracy							
Number of faulty responses (TT-err)	0.17	0.32	0.22	0.29	**0.51**	0.15	**0.66**
Number of omissions (TT-omis)	0.35	0.44	0.20	0.28	0.09	0.46	0.37
Standard Deviation of Lateral Position(SDLP)	0.23	0.47	0.27	0.37	0.21	0.29	0.49
*Mean reaction time dual task divided by mean reaction time single task (dual-to-single-task-ratio*, *DSR)*							
All stimuli (DSR-all)	**1.58**	**1.12**	0.70	0.18	0.46	**0.60**	**1.03**
Stimuli blind side (DSR-blind)	-	-	0.45	0.35	**0.68**	0.48	**1.31**
Stimuli seeing side (DSR-seeing)	-	-	0.03	0.03	0.01	0.02	0.00
**Obstacle course**							
Digit Score	**0.69**	0.37	0.28	0.15	0.33	0.04	0.37
Number of contacts	**1.04**	**1.10**	0.66	0.36	0.23	**0.67**	0.15
PPWS	**1.31**	**1.17**	0.43	0.15	0.21	0.00	0.21
**Questionnaires**							
NEI-VFQ-25 total score	-	-	0.65	0.17	0.16	**0.76**	**0.54**
IMQ total score	-	-	0.81	0.16	0.13	**0.74**	**0.55**
CVD total score	-	-	0.55	0.07	0.09	**0.71**	**0.56**

#### Basic scanning tests

At T1, both the training group and the waiting list control group showed significantly higher reaction times than the healthy controls on all conditions of the dot counting test and visual search tests (all *P* < 0.045), except for a non-significant difference between the waiting list control group and the healthy group for counting patterns with many dots (t(24.8) = 1.77, *P* = 0.089). Compared to the healthy control group, both the training group and the waiting list control group made more errors on some, but not all conditions of the dot counting test (HEALTHY vs. P-TRAINING: Dots-correct-all: t(44) = -2.92, *P* = 0.005; Dots-correct-few: *P* > 0.100; Dots-correct-many: t(44) = -2.98, *P* = 0.005; HEALTHY vs. P-WAITING: Dots-correct-all: t(32.1) = -2.09, *P* = 0.044; Dots-correct-few: t(26.1) = -1.74, *P* = 0.094; Dots-correct-many: t(46) = -1.99, *P* = 0.052). With regard to the accuracy rates on the two visual search tests, no significant differences were found between the healthy control group and the patient groups (HEALTHY vs. P-TRAINING: all *P* > 0.100; HEALTHY vs. P-WAITING: Par-err: F(26.6) = 1.74, *P* = 0.093; Par-omis: F (25.8) = 1.73, *P* = 0.096; Ser-err: F(46) = 1.73, *P* = 0.090; Ser-omis: F(46) = 1.70, *P* = 0.096). Analysis of effect sizes showed that the differences between the healthy control group and the patient groups regarding the reaction times in all three tests were exclusively medium or large. The differences in accuracy rates between the training group and the healthy control group were large for counting patterns with many dots and all trials. The remaining differences in accuracy rates between the training group and the healthy control group were small or negligible. Differences in accuracy rates between the waiting list control group and the healthy control group were all of medium size, except for a small effect in the number of omission errors on the serial search test.

#### Hazard perception test

The training group had significantly higher absolute (t(39) = 2.62, *P* = 0.012) and adapted error rates (t(39) = 2.56, *P* = 0.014) than the healthy control group, but the proportion of risky errors was not different. No significant differences were found between the waiting list control group and healthy control group (all *P* > 0.100). With regard to the effect sizes, the differences between the training group and the healthy control group were large for absolute and adapted error rate, while the difference for risk-index was negligible. The differences between the waiting list control group and the healthy control group were exclusively small.

#### Tracking Task

When steering and responding to peripheral stimuli simultaneously, both patient groups had longer average reaction times than the healthy control group (HEALTHY vs. P-TRAINING: t(46) = 5.17, *P* < 0.001; HEALTHY vs. P-WAITING: t(28.7) = 5.07, *P* ≤ 0.001). No significant group differences were found for SDLP and accuracy rates (all *P* > 0.100). While the healthy controls on average had equal reaction times for the single and dual task conditions (DSR = 1.00), patients had significantly higher DSRs (HEALTHY vs. P-TRAINING: t(32.4) = 5.34, *P* < 0.001; HEALTHY vs. P-WAITING: t(28.7) = 3.52, *P* = 0.001). The differences between both patient groups and the healthy control group for average reaction times and DSRs represented large effects. The group differences for SDLP and accuracy rates were all small or negligible.

#### Obstacle course

The training group had lower Digit Scores than the healthy control group (t(47) = -2.42, *P* = 0.020), while no significant difference was found between the waiting list control group and healthy control group. Compared to the healthy control group, both the training group and the waiting list control group touched more obstacles (HEALTHY vs. P-TRAINING: t(27.8) = 3.58, *P* = 0.001; HEALTHY vs. P-WAITING: t(24.6) = 3.67, *P* = 0.001) and had lower PPWS (HEALTHY vs. P-TRAINING: t(47) = -4.60, *P* < 0.001; HEALTHY vs. P-WAITING: t(46) = -4.04, *P* < 0.001). The difference between the training group and the healthy control group regarding the Digit Score was of medium size, while a small difference was found between the waiting list control group and the healthy control group. The group differences for number of contacts and PPWS all represented large effects.

### Training effects

#### Tests for visual functions

No significant group*time interaction effects were found for visual acuity, contrast sensitivity, and FFS (all *P* > 0.100). No significant changes between T1 and T2 were found for the training group (all *P* > 0.100). For the waiting list control group, the only parameter that changed significantly, was right eye visual acuity (F(1,47) = 4.15, *P* = 0.047; others: *P* > 0.100). All effect sizes for the group*time interaction effects and the changes within the patient groups, including the effect for right eye visual acuity, were small or negligible.

Analysis of changes in the border of the intact and blind visual field resulted in 52 comparisons between T1 and T2 for the training group (26 participants*2 eyes) and 46 for the control group. For the training group, no change was found in 27 cases, an enlargement of the visual field in 9 cases, a decrease in visual field in 12 cases, and in 4 cases part of the border shifted towards the seeing side, while another part shifted towards the blind side. For the waiting list control group, these values were not significantly different from the training group (values 15, 9, 14 and 8 respectively; χ(3) = 4.56, *P* = 0.207).

#### Reading tests

No significant group*time interaction effects were found for average reading speed and minimal readable text size on the Radner reading chart, nor for reading speed or correct answers on the standardized reading text (all *P* > 0.100). Similar results were obtained when analyses were performed separately for patients with left and right HVFD (all *P* > 0.100). For the training group, the number of correct answers after reading the text increased (t(23) = -2.15, *P* = 0.043; P-WAITING: *P* > 0.100). No other significant within-group changes between T1 and T2 were found (P-WAITING: text-reading speed: t(20) = -1.96, *P* = 0.064; others: *P* > 0.100). Regarding the analysis of effect sizes, only small and negligible effects were found for the group*time interactions and within-group differences.

#### Basic scanning tests

No significant group*time interaction effects were found for the reaction times or accuracy rates on the dot counting test and the visual search tests (all *P* > 0.100). Within the training group, no significant changes were found between T1 and T2 (Dots-correct-many: t(20) = -1.80, *P* = 0.087; other parameters all *P* > 0.100). The only significant change within the waiting list control group was a decrease in reaction time for counting patterns with few dots (t(22) = 2.33, *P* = 0.029; Par-target: t(22) = 1.82, *P* = 0.082; other parameters all *P* > 0.100). All group*time interaction effects and all differences between T1 and T2 within the patient groups were all of small or negligible size.

#### Hazard perception test

No group*time interaction effects were found for absolute error rate, adapted error rate or risk-index (all *P* > 0.100). Both the training group (absolute error rate: t(16) = 1.77, *P* = 0.096; adapted error rate: t(16) = 1.84, *P* = 0.085; risk-index: *P* > 0.100) and the waiting list control group (all *P* > 0.100) did not change significantly between T1 and T2 on these three parameters. The analysis of effect sizes revealed small effects for the group*time interaction. All changes between T1 and T2 were small for both patient groups, with the exception of a negligible effect for risk-index in the training group.

#### Tracking Task

**Fig 4:**
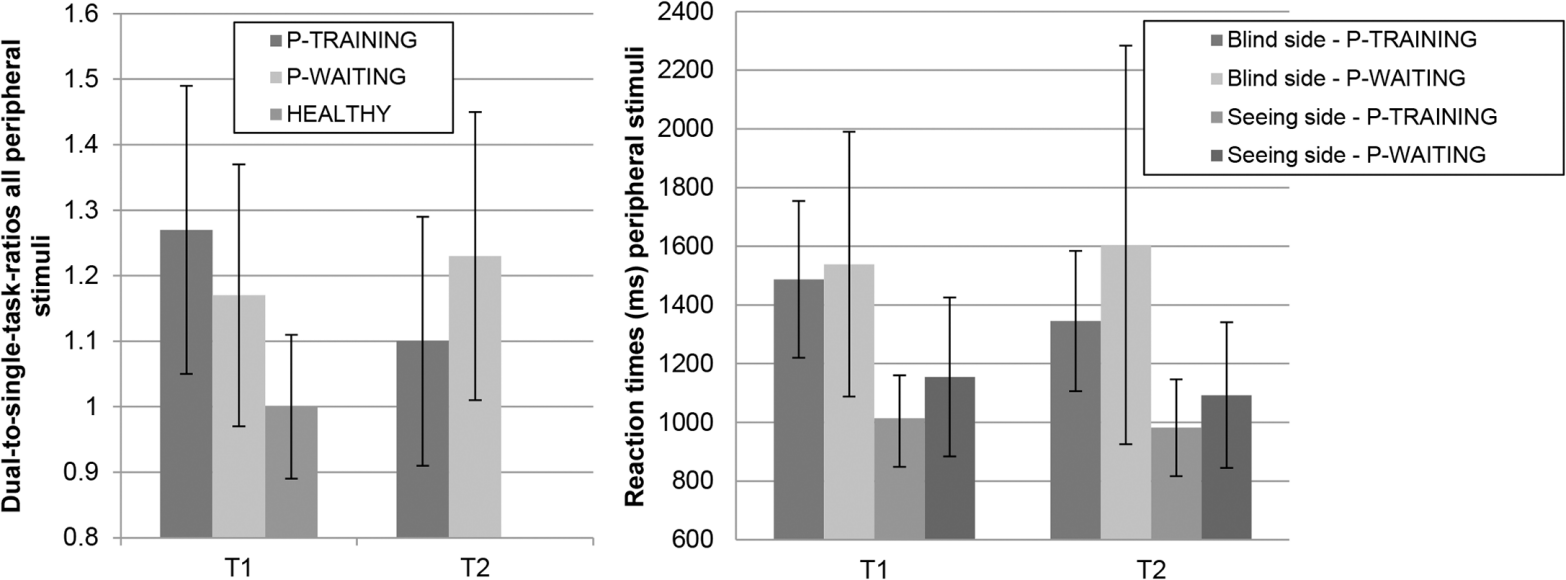
Results of the Tracking Task on T1 and T2 for the training group, waiting list control group and healthy control group (average ± SD).

Fig 4 presents data from the Tracking Task. A significant group*time interaction effect was found for the difference in reaction times between stimuli on the blind and seeing side (F(1,41) = 5.17, *P* = 0.028). However, neither the decrease in the training group, nor the increase in the waiting list control group was significant (both *P* > 0.100). No difference was found between the two patient groups regarding this parameter at T1 or T2 (both *P* > 0.100). No other significant group*time interactions were found (TT-RT-blind: F(1,41) = 3.45, *P* = 0.070; TT-err: F(1,42) = 3.41, *P* = 0.072; SDLP: F(1,41) = 3.49, *P* = 0.069; others: *P* > 0.100). A significant decrease in average reaction time (t(22) = 2.16, *P* = 0.042) and an almost significant decrease in reaction time for stimuli on the blind side (t(22) = 1.99, *P* = 0.059) were found for the training group, while no such effects were found for the waiting list control group (*P* > 0.100). On the other hand, the reaction times for stimuli on the seeing side were significantly reduced in the waiting list control group (t(20) = 2.17, *P* = 0.042), but not in the training group, noting that at T1, the training group reacted significantly faster on these stimuli than the waiting list control group. No significant within-group changes were found for accuracy rates or SDLP (all *P* > 0.100).

With regard to the DSRs, significant group*time interaction effects were found for stimuli on the blind side (F(1,40) = 6.71, *P* = 0.013) and the total number of stimuli (F(1,40) = 8.40, *P* = 0.006). These DSRs significantly decreased for the training group (DSR-blind: t(22) = 2.16, *P* = 0.042; DSR-all: t(22) = 3.35, *P* = 0.003), while no significant changes were found for the waiting list control group (*P* > 0.100). While the DSR-all was not significantly different for the two patient groups at T1, this difference just missed significance at T2 (t(41) = -1.95, *P* = 0.058). The training group started with a significantly higher DSR-blind compared to the waiting list control group at T1 (t(41) = 2.22, *P* = 0.032), while there was no significant difference at T2. No significant interaction effects or within group effects were found regarding the DSR for stimuli on the seeing side (*P* > 0.100).

The group*time interaction effect for the difference in reaction times between stimuli on the blind and seeing side was large. The changes within the patient groups, as well as the differences between the patient groups at T1 and T2 regarding this parameter were all of small size. The interaction effects for reaction times for stimuli on the blind side and the number of faulty responses were medium, while the remaining interaction effects had small or negligible sizes. The changes between T1 and T2 were all small for the training group and small or negligible for the waiting list control group. The interaction effects regarding DSR-all and DSR-blind were large. With regard to the DSR-all, the decrease in the training group was of medium size, while the increase in the waiting list control group was of negligible size. For the DSR-blind, both the decrease in the training group and the increase in the waiting list control group represented small changes. The group difference at T1 was small for DSR-all and medium for DSR-blind, while the group difference at T2 was medium for DSR-total and small for DSR-blind. All effects for DSR-seeing were negligible.

#### Obstacle course

**Fig 5:**
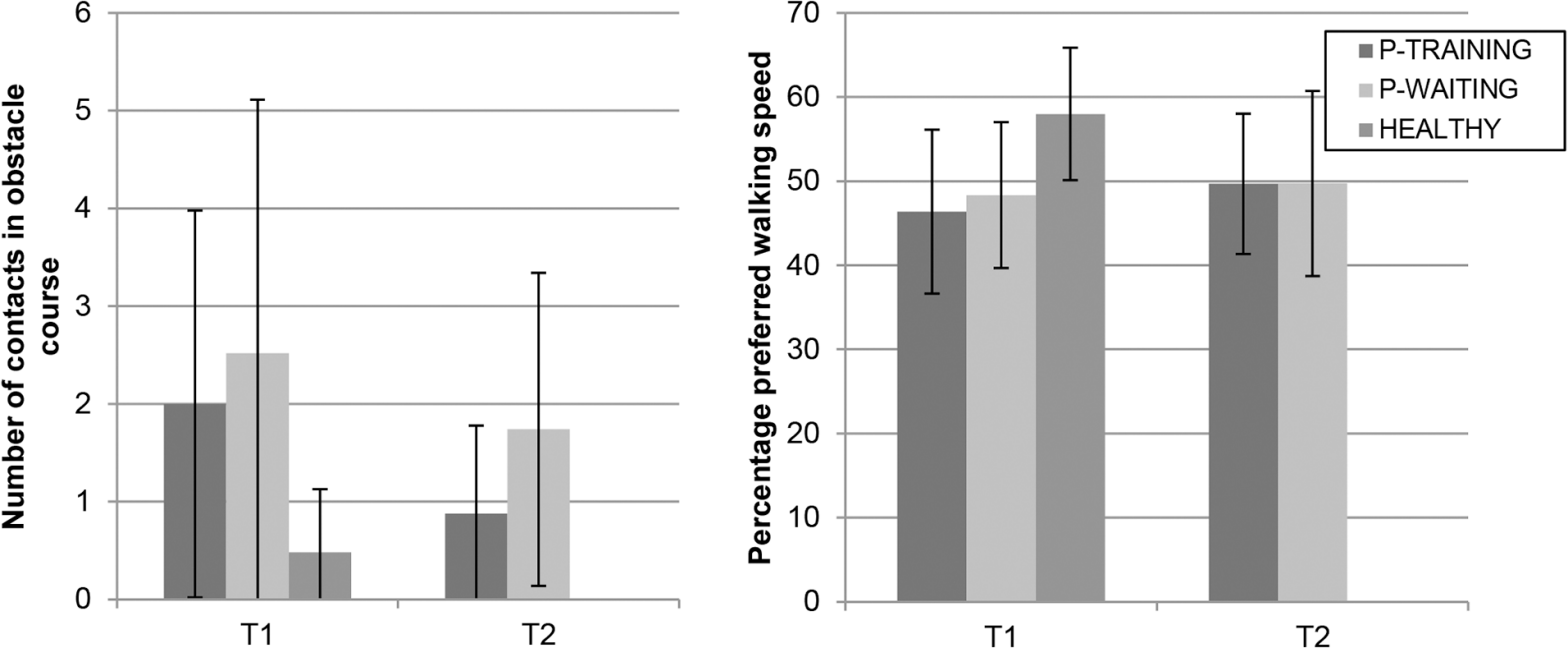
Number of contacts and Percentage Preferred Walking Speed in the obstacle course on T1 and T2 for the training group, waiting list control group and healthy control group (average ± SD).

Regarding performance in the obstacle course with cognitive load, no significant group*time interaction effects were found (*P* > 0.100). In the training group, however, number of contacts decreased (t(23) = 3.24, *P* = 0.004) and PPWS increased (t(23) = -2.12, *P* = 0.045) significantly after training, while no significant changes were found for the waiting list control group (contacts: t(22) = 1.73, *P* = 0.098; PPWS: *P* > 0.100; see Fig 5). Both patient groups showed no significant changes between T1 and T2 regarding the Digit Score (both *P* > 0.100). Analysis of the effect sizes showed that all interaction effects were small or negligible. Changes between T1 and T2 within the patient groups were all small or negligible, except for a decrease of medium effect size in the number of contacts for the training group.

#### Questionnaires

**Fig 6:**
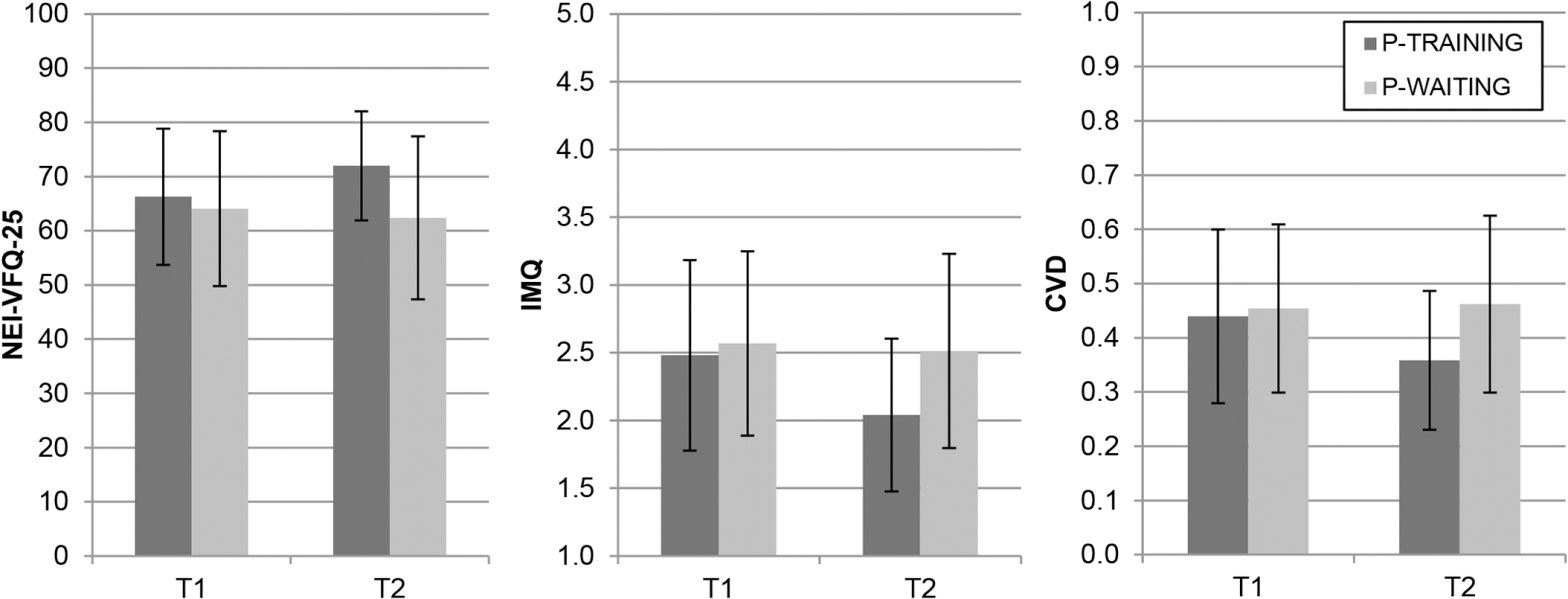
Questionnaire data on T1 and T2 for the training group and waiting list control group (average ± SD). Higher scores indicate less difficulties for NEI-VFQ-25 and more difficulties for IMQ and CVD.

Fig 6 presents the results of the questionnaires. The group*time interaction effects revealed that training decreased the self-reported impact of the HVFD on mobility and other visually related activities of daily life, while being on the waiting list did not. The interaction effects were significant for all three questionnaires (NEI-VFQ-25: F(1,46) = 9.74, *P* = 0.003; IMQ: F(1,46) = 8.00, *P* = 0.007; CVD: F(1,46) = 4.80, *P* = 0.034). The groups did not differ from each other at T1 (all *P* > 0.100). Between T1 and T2, the training group improved significantly (NEI-VFQ-25: t(25) = -3.32, *P* = 0.003; IMQ: t(25) = 4.13, *P* < 0.001; CVD: t(25) = 2.82, *P* = 0.009), while the waiting list control group did not (all *P* > 0.100). At T2, the training group scored significantly better than the waiting list control group (NEI-VFQ-25: t(37.7) = 2.59, *P* = 0.014; IMQ: t(47) = -2.58, *P* = 0.013; CVD: t(47) = -2.49, *P* = 0.017). The group*time interactions were of medium size for all three questionnaires. The group differences were negligible at T1. Improvements between T1 and T2 in the training group represented medium (VFQ, CVD) or large (IMQ) effects, while changes in the waiting list control group were exclusively negligible. At T2, the group differences were medium for all three questionnaires.

## Discussion

This is the first RCT to evaluate the effects of a compensatory scanning training that is based on a systematic horizontal scanning rhythm (IH-CST). Effects were measured on basic scanning tests, a hazard perception test, an obstacle course and questionnaires on experienced difficulties in daily life, all of which were different from the training exercises. Furthermore, visual functions and reading performance were assessed before and after training. Performance of patients on the scanning and mobility-related measures at first assessment was compared to performance of a healthy control group, and performance prior to and following training was compared to performance of a patient waiting list control group. The key findings are summarized in Table 4.

**Table 4:** Key findings of the study.

	Comparing the patients to the healthy control group on T1	Comparing T1 and T2 for the training group and waiting list control group
Tests for visual functions		No evidence was found for changes in visual acuity, contrast sensitivity, and visual field size in both groups, except for a small, but significant improvement in right eye visual acuity for the waiting list control group.
Reading tests		No evidence was found for changes in reading performance in both groups, except for a small, but significant increase in correct answers to the questions about the text in the training group.
Basic scanning tests	Patients had higher reaction times on the dot counting test and the visual search tests. Patients made more errors when counting dots (mainly when counting many dots) compared to the healthy control group, while no significant differences were found for accuracy rates in the visual search tests.	No evidence was found for changes in performance on the dot counting test and the visual search tests in both groups, except for a small, but significant decrease in reaction times for counting patterns with few dots in the waiting list control group.
Hazard perception test	The training group had significantly higher absolute and adapted error rates than the healthy control group, but the proportion of risky errors was not different. No significant differences were found between the waiting list control group and healthy control group.	No evidence was found for changes in performance in both groups.
Tracking Task	Patients needed significantly more time to respond to the peripheral stimuli, while no significant differences were found for accuracy rates on the peripheral task and performance on the central task (SDLP). While the healthy controls on average had equal reaction times for the single and dual task conditions, patients had significantly higher in dual-to-single-task-ratios, meaning that the reaction times suffered from the dual task (i.e. the central task).	In the dual task condition, the difference in reaction times between stimuli on the blind and seeing side decreased for the training group, while it increased for the waiting list control group. The average reaction times decreased significantly in the training group, but not in the waiting list control group. These improvements in the training group did not result in higher reaction times stimuli on the seeing side, nor did it affect performance on the central and peripheral task. After training, patients seemed to be troubled less by an additional central task, according to a decrease in dual-to-single-task-ratios for stimuli on the blind side, while no effect was found for the waiting list control group.
Obstacle course	Patients touched more obstacles and had lower PPWS then the healthy control participants. Compared to the healthy control group, the training group had lower Digit Scores, but no significant difference was found for the waiting list control group.	In the training group, the number of contacts decreased and PPWS increased, while no significant changes were found for the waiting list control group. This improvement in the training group did not cause a decline in performance on the cognitive task that was performed during walking.
Questionnaires		According to the questionnaire data, the detrimental impact of HVFD on mobility in daily life decreased considerably in the training group, but not in the waiting list control group. Patients in the training group reported that after training, they performed more mobility-related activities with less difficulty, and they felt that their vision-related quality of life had improved.

Compared to healthy control participants, who were matched with the total patient group on age and level of education, patients with HVFD needed more time when counting dot patterns or searching for targets among distractors, needed more time to detect peripheral stimuli, especially in a dual task condition, and showed more difficulty in avoiding obstacles and maintaining preferred walking speed when walking through an obstacle course while performing a cognitive task. Evidence was found for an improvement after IH-CST on all of these tests, except for the basic scanning tests (dot counting and visual search). These results are in agreement with results from previous studies showing that patients with HVFD perform worse than healthy control participants on visual search tasks [54] and peripheral detection in dynamic environments [55,56].

According to the questionnaire data, the detrimental impact of HVFD on mobility in daily life decreased considerably after training, as indicated by significant effects, mainly of medium size. Participants reported that after training, they performed more mobility-related activities with less difficulty, and they felt that their vision-related quality of life had improved. These self-reported improvements were accompanied by improvements on some, but not all, objective outcome measures. The improvements were not mediated by improvements in visual functions such as an increased visual field, since no considerable changes regarding the visual functions were found between T1 and T2.

Training decreased the difference in reaction times between stimuli on the blind and seeing side when patients performed a central task simultaneously, as indicated by a large and significant group*time interaction effect on the Tracking Task, although within-group changes were small and not significant. This improvement does not seem to come at the expense of the absolute reaction times for stimuli on the blind side or for stimuli on the seeing side. Furthermore, correct detection of the peripheral stimuli and performance on the central task were not negatively affected. A small, but significant within-group effect was found for a decrease in overall reaction times for the training group only. Furthermore, the reaction times for stimuli on the blind side specifically decreased in the training group and not in the waiting list control group, as indicated by an interaction effect of medium size. These results suggest that after training, patients with HVFD spread their visual attention more evenly across the left and right side, while still paying attention to the situation in front. In mobility situations in particular, it is of high importance that information from both the left and right side as well as from what is happening in front of the person, is being perceived and processed efficiently.

After training, patients with HVFD seem to be troubled less by an additional central task, as indicated by large and significant group*time interaction effects for the dual-to-single-task-ratios for the total number of peripheral stimuli and for stimuli on the blind side specifically (Tracking Task). Patients who received training showed significant decreases of medium and small size respectively for these two dual-to-single-task-ratios. Results of the obstacle course showed that after training, patients touched fewer obstacles when walking through a standardized obstacle course and performing a cognitive task simultaneously. This improvement was not at the expense of performance on the cognitive task or walking speed. In fact, PPWS increased significantly after training, meaning that the impact of obstacles and cognitive load on walking speed decreased, although the effect was only small. These findings suggest that compensation in dual task conditions becomes easier after IH-CST. Although patients with HVFD often know they should compensate by looking towards the blind side, this may be very hard in dual task situations such as having a conversation while walking, because the second task limits the attentional capacity available for compensatory scanning or because compensatory scanning efforts impair performance on the second task. The present findings suggest that after training, the skill of applying the systematic scanning rhythm was automatized, at least to some extent, increasing free attentional capacity for other tasks.

No effects of training were found for the dot counting test and the visual search tests. Apparently, the scanning rhythm as taught in the IH-CST was not very helpful during tasks that require visual counting or visual search. When searching for a predefined target in a complex display, such as a shelf in a shop, a large saccade towards the blind side may help to get a first overview, but in case of complex displays requiring serial search, every object or feature has to be watched separately. A spatially organized search pattern might then be preferred. As opposed to the IH-CST, the CST programs in the previous RCT studies [14,16,19–21] were all based on searching for targets among distractors and they consistently found improvements on visual search tests after training.

With regard to the accuracy scores of the hazard perception test, no evidence for an effect of training was found. As suggested by Aimola and colleagues [14], who also failed to find an effect of CST on a similar test, hazard perception supposedly requires skills beyond eye-movement strategies. Decisions on which action to perform in a specific situation may rely on other factors, such as driving experience and personality traits. In the current study, participants were likely to have perceived the whole picture after the presentation time of eight seconds, which is a long period even in the case of inefficient scanning. Future research on eye tracking data could include analysis of the time participants need before they fixate on areas of interest presenting potential hazards.

No effect of training on reading performance was found, except for a small, but significant increase in correct answers after reading a standardized text in the training group. The absence of an effect on reading speed is not surprising, since small and precise saccades are necessary during reading, while the scanning strategy as applied in the IH-CST consists of large saccades towards the far periphery. These findings correspond to the results of previous RCTs on CST [14,16,19–21]. Reading performance was assessed in all four studies, but an improvement in reading was only found after training programs including reading exercises [14,21].

In summary, the present findings show that the IH-CST, which is based on learning to apply a top-down, systematic horizontal scanning strategy, specifically improves detection of peripheral stimuli in mobility situations, without limiting, or even improving simultaneously performed activities. No evidence was found for improvements on dot counting, visual search or reading. The relative specificity of the training effect is in accordance with the findings of previous RCT studies on visual search training, which mainly found effects on tests similar to the exercises practiced during training. Schuett [21], for example, found that CST with visual search exercises only improved visual search while reading training only improved reading performance, indicating that the training effects were specific and task-dependent. Aimola and colleagues [14] also reported that the effects of CST were restricted to tasks that resembled the training exercises.

In contrast to these previous studies, the present study found evidence for a transfer of training effects to activities that were different from the exercises applied during training. However, IH-CST only improved mobility-related activities in which detection of peripheral stimuli is important, while no improvement was found on tests that require other visual skills, such as reading and, apparently, visual search. The finding that CST based on visual search exercises caused specific improvements on visual search tests, while the IH-CST based on a systematic scanning rhythm did not cause such an effect, also suggests that different scanning strategies are required for visual search as for detecting peripheral information. Hardies and colleagues [57] found evidence for different compensatory strategies being helpful for different types of scanning tasks.

This is the first RCT to find an improvement of CST on mobility performance. Of all studies examining the effects of CST with exercises of horizontal scanning using a within-subject design, only Tant and colleagues [31] included mobility assessments and they found an improvement in visual-spatial performance during driving. The study of Aimola and colleagues [14] is the only RCT besides the present study that analyzed the transfer of the training effect to objective mobility-related tests. However, their CST was based on unsupervised reading training and CST with visual search exercises and no improvement was found for walking speed in an obstacle course. This suggests that the training of horizontal scanning strategies has a higher potential for improving mobility in daily life than the training of visual search strategies. This suits the idea that engaging in traffic does not so much rely on searching for specific targets, while early detection of all relevant objects is essential for anticipation in the dynamic traffic situations. Furthermore, the improvement on mobility-related tests as found in the present study suggests high importance of certain training characteristics, such as a specific top-down scanning strategy, training exercises with targets in the far periphery (beyond 40 degrees from the midline), feedback of a therapist, and inclusion of exercises in daily life mobility situations, none of which were included in the training examined by Aimola and colleagues [14]. At present, the data cannot tell which of these characteristics are most important or if it is the combination of these characteristics that is valuable. Furthermore, the present data cannot tell to what degree the face-to-face training and the homework assignments have contributed to the improvements.

A few remarks to the present study: Although an RCT design with a waiting list control group controls for maturation and testing effects, the risk of placebo effects remains. The degree to which the intensive attention and support from the therapist influenced performance of the patients cannot be determined. The specificity of the present results, however, suggests that the improvements are not merely non-specific placebo effects. The mobility-related activities improved specifically, even though these activities were different from the training exercises. The small sample sizes might have prohibited the detection of further effects, however, the exclusively negligible and small sizes of the effects on dot counting and visual search indicate that there is presumably no effect of the IH-CST on these tasks. Therefore, the inclusion of larger sample sizes will presumably also not reveal such effects. It cannot be not ruled out, however, that a higher number of training sessions could have resulted in improvements on the other tests, such as the dot counting test. Another factor that possibly influenced the outcome of the training are the differences in performance at T1 between the two patient groups that was found for some tests. This might have contributed to failure in detecting certain effects. The finding that none of the significant group*time interaction effects could be fully explained by a significant between-group difference at T1 argues against type I errors. Unfortunately, analyses of eye tracking data could not be performed in the present study. Including analyses of eye tracking data in future studies might provide more insight into the question which scanning mechanisms are specifically helpful for different types of visual tasks. Another suggestions for future research on the CST is to examine whether specific components of the IH-CST protocol are more useful than others, which might be related to individual differences in patient characteristics or rehabilitation goals.

In conclusion, the IH-CST trains patients with HVFD to apply a systematic scanning rhythm, which helps them to compensate for their visual field defect in specific tasks with specific demands, mainly detection of peripheral stimuli in mobility situations. This skill is practiced under supervision of a therapist in a step-by-step manner, from simple scanning exercises to practicing the scanning rhythm in high-demanding mobility situations of daily life. This skill is automatized as much as possible, in order to benefit in daily life situations, which are often dual task situations. After training, participants indeed felt less impaired in mobility situations. These self-reported improvements were accompanied by improvements in detecting peripheral stimuli and avoiding obstacles during walking, especially in dual task situations in which a second task limits the attentional capacity available for compensatory scanning. The results indicate that reading, but also searching for a target amongst distractors, requires different compensatory scanning mechanisms than fast detection of peripheral stimuli in mobility situations. In previous literature, the terms visual search and visual exploration have been used for a wide range of different visual tasks. Professionals involved in the research, development and application of scanning training for HVFD patients are advised to consciously reflect on which type of compensatory scanning strategy is appropriate for the specific activity they aim to examine or improve.

## Supporting Information

S1 Checklist CONSORT Checklist. (DOC)

S2 Checklist TIDieR checklist. (DOCX)

S1 Protocol Original study protocol. (DOC)

S1 File Individual-level data. (XLSX)
