# Correction: The Effects of Compensatory Scanning Training on Mobility in Patients with Homonymous Visual Field Defects: A Randomized Controlled Trial

**DOI:** 10.1371/journal.pone.0165863

**Published:** 2016-10-27

**Authors:** Gera A. de Haan, Bart J. M. Melis-Dankers, Wiebo H. Brouwer, Oliver Tucha, Joost Heutink

There is an error in the last sentence of the “Hazard perception test” section in the “Assessments” section of the Material and Methods. The correct sentence is: The risk-index was defined by the proportion of risky answers (risk-index = (2*very risky responses + risky responses) / adapted error rate).

There is an error in Table 2. The values in the row “Contrast sensitivity” are incorrect. Please see the corrected Table 2 here.

**Table 2:** Test scores (mean ± SD).

	Training group			Waiting list control group			Healthy control group	
	n	T1	T2	n	T1	T2	n	T1
		(Before training)	(After training)		(Early pre-assessment)	(Before training)		
**Tests for visual functions**								
Visual acuity right eye (VOD)	26	0.90 ± 0.28	0.91 ± 0.29	23	0.90 ± 0.25	**0.99 ± 0.26** b	-	
Visual acuity left eye (VOS)	26	0.97 ± 0.26	0.97 ± 0.25	23	0.90 ± 0.26	0.92 ± 0.26	-	
Contrast sensitivity	26	1.94± 0.16	1.97 ± 0.08	23	1.93 ± 0.13	1.90 ± 0.19	-	
Functional Field Score (FFS)	26	**57.92 ± 7.80** c	57.75 ± 6.74	23	63.79 ± 11.41	62.58 ± 11.13	-	
**Reading tests**								
Radner average reading speed(wpm)	24	153 ± 31	159 ± 33	21	146 ± 41	147 ± 34	-	-
Minimal readable text size (LogRad)	24	0.08 ± 0.15	0.09 ± 0.12	21	0.07 ± 0.10	0.07 ± 0.12	-	-
Text reading speed (wpm)	23	133 ± 23	136 ± 27	21	125 ± 31	135 ± 35	-	-
Text correct answers	24	1.46 ± 0.66	**1.79 ± 0.42** b	21	1.62 ± 0.59	1.67 ± 0.48	-	-
**Basic scanning tests**								
*Dot counting test*								
Reaction times (ms)								
All trials (Dots-RT-all)	21	**8904 ± 3434** a	8515 ± 3812	23	**9293 ± 5142** a	8224 ± 3201	25	6631 ± 1496
Few dots (Dots-RT-few)	21	**5272 ± 2315** a	4834 ± 2012	23	**5115 ± 1923** a	**4542 ± 1408**^b^	25	3214 ± 818
Many dots (Dots-RT-many)	21	**12495 ± 4874** a	12207 ± 6175	23	13471 ± 8993	11942 ± 5376	25	10048 ± 2347
Proportion correct answers								
All trials (Dots-correct-all)	21	**0.72 ± 0.17** a	0.75 ± 0.18	23	**0.73 ± 0.24** a	0.70 ± 0.28	25	0.84 ± 0.12
Few dots (Dots-correct-few)	21	0.93 ± 0.09	0.91 ± 0.20	23	0.88 ± 0.22	0.85 ± 0.28	25	0.96 ± 0.07
Many dots (Dots-correct-many)	21	**0.50 ± 0.29** a	0.59 ± 0.29	23	0.57 ± 0.31	0.56 ± 0.34	25	0.72 ± 0.21
*Parallel search test*								
Reaction times (ms)								
All trials (Par-RT-all)	21	**2369 ± 785** a	2224 ± 838	23	**2183 ± 516** a	2140 ± 545	25	1196 ± 367
Target present (Par-RT-target)	21	**1539 ± 562** a	1422 ± 425	23	**1498 ± 425** a	1416 ± 389	25	996 ± 260
Target absent (Par-RT-notarget)	21	**3199 ± 1027** a	3027 ± 1286	23	**2868 ± 768** a	2861 ± 793	25	1396 ± 504
Accuracy								
Total number of errors (Par-err)	20	0.45 ± 0.83	0.40 ± 0.50	23	1.35 ± 2.27	0.83 ± 1.56	25	0.48 ± 0.77
Number of omissions (Par-omis)	20	0.20 ± 0.41	0.25 ± 0.44	23	1.09 ± 2.04	0.70 ± 1.55	25	0.32 ± 0.63
*Serial search test*								
Reaction times (ms)								
All trials (Ser-RT-all)	21	**5563 ± 1592** a	5258 ± 1541	23	**4998 ± 2039** a	5196 ± 2269	25	3498 ± 1337
Target present (Ser-RT-target)	21	**3855 ± 1472** a	3607 ± 1031	23	**3602 ± 1709** a	3676 ± 1725	25	2600 ± 1321
Target absent (Ser-RT-notarget)	21	**7270 ± 1891** a	6909 ± 2244	23	**6394 ± 2607** a	6715 ± 3123	25	4395 ± 1474
Accuracy								
Total number of errors (Ser-err)	21	1.14 ± 1.59	1.57 ± 1.94	23	1.78 ± 2.32	1.78 ± 1.93	25	0.84 ± 1.38
Number of omissions (Ser-omis)	21	0.95 ± 1.12	1.48 ± 1.81	23	1.61 ± 2.04	1.74 ± 1.94	25	0.76 ± 1.39
**Hazard perception test**								
Absolute error rate	17	**10.41 ± 2.43** a	9.24 ± 2.33	22	9.32 ± 2.77	8.68 ± 3.00	24	8.50 ± 2.21
Adapted error rate	17	**11.65 ± 3.16** a	10.12 ± 2.69	22	10.27 ± 3.34	9.55 ± 3.35	24	9.21 ± 2.89
Risk-index	17	0.73 ± 0.16	0.74 ± 0.13	22	0.69 ± 0.21	0.74 ± 0.20	24	0.76 ± 0.18
**Tracking Task**								
*Dual task condition*								
Reaction times (ms)								
All stimuli (TT-RT-all)	23	**1200 ± 195** a	**1109 ± 155** b	21	**1293 ± 286** a	1271 ± 341	25	943 ± 147
Stimuli blind side (TT-RT-blind)	23	1487 ± 267	1345 ± 239	21	1539 ± 451	1605 ± 679	-	-
Stimuli seeing side (TT-RT-seeing)	23	**1014 ± 146** c	982 ± 165	21	1155 ± 271	**1093 ± 248** b	-	-
Stimuli blind side-stimuli seeing side	23	473 ± 263	362 ± 246	21	384 ± 313	**512 ± 514** e	-	-
Accuracy								
Number of faulty responses (TT-err)	24	0.67 ± 0.92	0.92 ± 1.02	21	1.19 ± 1.12	0.76 ± 1.14	25	0.84 ± 1.07
Number of omissions (TT-omis)	24	0.29 ± 1.00	0.08 ± 0.28	21	0.38 ± 1.12	0.57 ± 1.54	25	0.04 ± 0.20
Standard Deviation of Lateral Position (SDLP)	23	48.09 ± 9.94	50.30 ± 10.27	21	50.19 ± 10.55	47.31 ± 10.59	25	46.14 ± 6.50
*Mean reaction time dual task divided by mean reaction time single task (dual-to-single-task-ratio*, *DSR)*								
All stimuli (DSR-all)	23	**1.27 ± 0.22** a	**1.10 ± 0.19** b	20	**1.17 ± 0.20** a	**1.23 ± 0.22** e	25	1.00 ± 0.11
Stimuli blind side (DSR- blind)	23	**1.54 ± 0.43** c	**1.29 ± 0.38** b	20	1.29 ± 0.31	**1.53 ± 0.63** e	-	-
Stimuli seeing side (DSR- seeing)	23	1.07 ± 0.20	1.06 ± 0.19	20	1.07 ± 0.22	1.06 ± 0.18	-	-
**Obstacle course**								
Digit Score	24	**0.60 ± 0.31** a	0.66 ± 0.31	23	0.70 ± 0.27	0.65 ± 0.29	25	0.79 ± 0.24
Number of contacts	24	**2.00 ± 1.98** a	**0.88 ± 0.90** bd	23	**2.52 ± 2.59** a	1.74 ± 1.60	25	0.48 ± 0.65
PPWS	24	**46.36 ± 9.75** a	**49.68 ± 8.35** b	23	**48.33 ± 8.67** a	49.72 ± 11.02	25	57.98 ± 7.88
**Questionnaires**								
NEI-VFQ-25 total score	26	66.30 ± 12.56	**71.98 ± 10.07** bd	23	64.10 ± 14.30	**62.39 ± 15.06**^e^	-	-
IMQ total score	26	2.48 ± 0.70	**2.04 ± 0.56** bd	23	2.57 ± 0.68	**2.51 ± 0.72** e	-	-
CVD total score	26	0.44 ± 0.16	**0.36 ± 0.13** bd	23	0.45 ± 0.15	**0.46 ± 0.16** e	-	-

^a^significant difference between the patient group and healthy control group at T1 (independent samples t-test, two-sided *P*-value < 0.050).

^b^significant within-group difference between T1 and T2 (matched pairs t-test, two-sided *P*-value < 0.050).

^c^significant difference between training group and waiting list control group at T1 (independent samples t-test, two-sided *P*-value < 0.050).

^d^significant difference between training group and waiting list control group at T2 (independent samples t-test, two-sided *P*-value < 0.050).

^e^significant Group (training vs. waiting list control) * Time (T1 vs. T2) interaction effect (GLM Repeated Measures, *P*-value < 0.050).

There is an error in Table 3. The values in the row “Contrast sensitivity” are incorrect. Please see the corrected Table 3 here.

**Table 3:** Effect sizes for within-group and between-group comparisons (Cohen’s d [52]) and group*time interactions (d_ppc2_ as described by Morris [51]).

	Training vs. healthy at T1	Waiting list vs. healthy at T1	T1 vs. T2 for Training group	T1 vs. T2 for Waiting list group	Training vs. Waiting list at T1	Training vs. Waiting list at T2	Time*group Interaction
**Tests for visual functions**							
Visual acuity right eye (VOD)	-	-	0.08	0.45	0.01	0.27	0.29
Visual acuity left eye (VOS)	-	-	0.02	0.09	0.27	0.19	0.08
Contrast sensitivity	-	-	0.27	0.22	0.06	0.50	0.43
Functional Field Score (FFS)	-	-	0.04	0.24	0.61	**0.53**	0.11
**Reading tests**							
Radner average reading speed (wpm)	-	-	0.23	0.03	0.20	0.36	0.13
Minimal readable text size (LogRad)	-	-	0.09	0.00	0.09	0.18	0.06
Text reading speed (wpm)	-	-	0.13	0.43	0.32	0.03	0.27
Text correct answers	-	-	0.44	0.06	0.26	0.27	0.44
**Basic scanning tests**							
*Dot counting task*							
Reaction times (ms)							
All trials (Dots-RT-all)	**0.89**	**0.72**	0.13	0.29	0.09	0.08	0.15
Few dots (Dots-RT-few)	**1.23**	**1.31**	0.28	0.49	0.07	0.17	0.06
Many dots (Dots-RT-many)	**0.66**	**0.53**	0.06	0.23	0.13	0.05	0.17
Proportion correct answers							
All trials (Dots-correct-all)	**0.87**	**0.62**	0.23	0.18	0.05	0.17	0.24
Few dots (Dots-correct-few)	0.41	**0.52**	0.10	0.20	0.30	0.23	0.04
Many dots (Dots-correct-many)	**0.88**	**0.58**	0.39	0.07	0.23	0.09	0.32
*Parallel search task*							
Reaction times (ms)							
All trials (Par-RT-all)	**1.97**	**2.22**	0.14	0.19	0.28	0.12	0.15
Target present (Par-RT-target)	**1.28**	**1.44**	0.21	0.38	0.08	0.01	0.07
Target absent (Par-RT-notarget)	**2.29**	**2.29**	0.11	0.02	0.37	0.16	0.18
Accuracy							
Total number of errors (Par-err)	0.04	**0.52**	0.05	0.25	**0.51**	0.36	0.26
Number of omissions (Par-omis)	0.22	**0.52**	0.10	0.22	**0.58**	0.38	0.28
*Serial search task*							
Reaction times (ms)							
All trials (Ser-RT-all)	**1.42**	**0.88**	0.16	0.15	0.31	0.03	0.27
Target present (Ser-RT-target)	**0.90**	**0.66**	0.18	0.07	0.16	0.05	0.20
Target absent (Ser-RT-notarget)	**1.71**	**0.95**	0.14	0.18	0.38	0.07	0.29
Accuracy							
Total number of errors (Ser-err)	0.20	**0.50**	0.16	0.00	0.32	0.11	0.21
Number of omissions (Ser-omis)	0.15	0.49	0.24	0.08	0.40	0.14	0.24
**Hazard perception test**							
Absolute error rate	**0.83**	0.33	0.43	0.26	0.42	0.21	0.20
Adapted error rate	**0.81**	0.34	0.45	0.30	0.42	0.19	0.24
Risk-index	0.14	0.36	0.05	0.26	0.25	0.01	0.25
**Tracking Task**							
*Dual task condition*							
Reaction times (ms)							
All stimuli (TT-RT-all)	**1.49**	**1.58**	0.45	0.13	0.38	**0.62**	0.28
Stimuli blind side (TT-RT-blind)	-	-	0.42	0.16	0.14	**0.52**	**0.56**
Stimuli seeing side (TT-RT-seeing)	-	-	0.21	0.47	**0.66**	**0.53**	0.14
Stimuli blind side—stimuli seeing side	-	-	0.33	0.30	0.31	0.38	**0.82**
Accuracy							
Number of faulty responses (TT-err)	0.17	0.32	0.22	0.29	**0.51**	0.15	**0.66**
Number of omissions (TT-omis)	0.35	0.44	0.20	0.28	0.09	0.46	0.37
Standard Deviation of Lateral Position (SDLP)	0.23	0.47	0.27	0.37	0.21	0.29	0.49
*Mean reaction time dual task divided by mean reaction time single task (dual-to-single-task-ratio*, *DSR)*							
All stimuli (DSR-all)	**1.58**	**1.12**	**0.70**	0.18	0.46	**0.60**	**1.03**
Stimuli blind side (DSR-blind)	-	-	0.45	0.35	**0.68**	0.48	**1.31**
Stimuli seeing side (DSR-seeing)	-	-	0.03	0.03	0.01	0.02	0.00
**Obstacle course**							
Digit Score	**0.69**	0.37	0.28	0.15	0.33	0.04	0.37
Number of contacts	**1.04**	**1.10**	**0.66**	0.36	0.23	**0.67**	0.15
PPWS	**1.31**	**1.17**	0.43	0.15	0.21	0.00	0.21
**Questionnaires**							
NEI-VFQ-25 total score	-	-	**0.65**	0.17	0.16	**0.76**	**0.54**
IMQ total score	-	-	**0.81**	0.16	0.13	**0.74**	**0.55**
CVD total score	-	-	**0.55**	0.07	0.09	**0.71**	**0.56**

There are errors in S1 File. Please see the corrected S1 File here.

## Supporting Information

S1 File Individual-level data. (XLSX)
