# Supplementary material for: The Effects of Compensatory Scanning Training on Mobility in Patients with Homonymous Visual Field Defects: A Randomized Controlled Trial
Source: PLoS One. 2015 Aug 14;10(8):e0134459. doi: 10.1371/journal.pone.0134459 (PMC4537273; doi:10.1371/journal.pone.0134459)
Supplement: S1 Protocol — (DOC) [file pone.0134459.s003.doc]

**STUDY PROTOCOL**

**approved by ethics committee UMCG**

The effects of Scanning Compensatory Therapy for patients with homonymous visual field defects – a randomised controlled trial

**15-07-2010**

The effects of Scanning Compensatory Therapy for patients with homonymous visual field defects – a randomised controlled trial

| Date |  |
| --- | --- |
| Project leaders | W.H. Brouwer PhD  Dep. of Clinical and Developmental Neuropsychology, University of Groningen, Groningen, The Netherlands  Department of Neurology, University Medical Center Groningen, Groningen, The Netherlands  +31 50 3614663  W.H.Brouwer@rug.nl  J.H.C. Heutink PhD  Dep. of Clinical and Developmental Neuropsychology, University of Groningen, Groningen, The Netherlands  +31 50 3611805  j.h.c.heutink@rug.nl  Royal Dutch Visio: Centre of Expertise for Blind and Partially Sighted People, Haren, The Netherlands  +31 50 5997100  joostheutink@visio.org  B.J.M. Melis-Dankers PhD  Royal Dutch Visio: Centre of Expertise for Blind and Partially Sighted People, Haren, The Netherlands  Department of Ophthalmology, University Medical Center Groningen, Groningen, The Netherlands  +31 88 585 70 00  BartMelis@visio.org |
| Principal investigator | G.A. de Haan MSc  Dep. of Clinical and Developmental Neuropsychology, University of Groningen, Groningen, The Netherlands  +31 50 3614664  G.A.de.Haan@rug.nl  geradehaan@visio.org |
| Collaboration | University of Groningen, Groningen, The Netherlands  Royal Dutch Visio, Haren, The Netherlands  UMCG Ophthalmology  UMCG Neurology |

**PROTOCOL SIGNATURE SHEET**

| **Name** | **Signature** | **Date** |
| --- | --- | --- |
| **For non-commercial research,**  **Head of Department:**  **O.M.Tucha** |  |  |
| **Principal investigator**  **G.A. de Haan MSc.** |  |  |

**Table of contents**

SUMMARY [5](#__RefHeading___Toc408902476)

1. INTRODUCTION [7](#__RefHeading___Toc408902477)

2. OBJECTIVES AND RESEARCH QUESTIONS [9](#__RefHeading___Toc408902478)

3. DESIGN [10](#__RefHeading___Toc408902479)

4. STUDY POPULATION [12](#__RefHeading___Toc408902480)

4.1. Population [12](#__RefHeading___Toc408902481)

4.2. Inclusion criteria [12](#__RefHeading___Toc408902482)

4.3. Exclusion criteria [13](#__RefHeading___Toc408902483)

4.4. Sample size [14](#__RefHeading___Toc408902484)

5. INTERVENTION [15](#__RefHeading___Toc408902485)

5.1. Compensatory Scanning Training [15](#__RefHeading___Toc408902486)

6. METHODS [16](#__RefHeading___Toc408902487)

6.1. Study parameters [16](#__RefHeading___Toc408902488)

6.1.1. Primary outcome measures [16](#__RefHeading___Toc408902489)

6.1.2. Secondary outcome measures [16](#__RefHeading___Toc408902490)

6.1.3. Other measures [18](#__RefHeading___Toc408902491)

6.2. Selection [19](#__RefHeading___Toc408902492)

6.3. Study procedures [20](#__RefHeading___Toc408902493)

6.4. Withdrawal of individual subjects [20](#__RefHeading___Toc408902494)

6.5. Replacement of individual subjects after withdrawal [20](#__RefHeading___Toc408902495)

6.6. Follow-up of subjects withdrawn from treatment [20](#__RefHeading___Toc408902496)

7. SAFETY REPORTING [21](#__RefHeading___Toc408902497)

7.1. Section 10 WMO event [21](#__RefHeading___Toc408902498)

7.2. Adverse and serious adverse events [21](#__RefHeading___Toc408902499)

7.3. Follow-up of adverse events [21](#__RefHeading___Toc408902500)

8. STATISTICAL ANALYSIS [22](#__RefHeading___Toc408902501)

8.1. Descriptives [22](#__RefHeading___Toc408902502)

8.2. Univariate analysis [22](#__RefHeading___Toc408902503)

8.3. Multivariate analysis [22](#__RefHeading___Toc408902504)

9. ETHICAL CONSIDERATIONS [23](#__RefHeading___Toc408902505)

9.1. Regulation statement [23](#__RefHeading___Toc408902506)

9.2. Inclusion and consent [23](#__RefHeading___Toc408902507)

9.3. Justification of burden on participants [23](#__RefHeading___Toc408902508)

9.4. Insurance [23](#__RefHeading___Toc408902509)

9.5. Compensation [23](#__RefHeading___Toc408902510)

10. ADMINISTRATIVE ASPECTS AND PUBLICATION [24](#__RefHeading___Toc408902511)

10.1. Handling and storage of data and documents [24](#__RefHeading___Toc408902512)

10.2. Amendments [24](#__RefHeading___Toc408902513)

10.3. Annual progress report [24](#__RefHeading___Toc408902514)

10.4. End of study report [24](#__RefHeading___Toc408902515)

10.5. Publication policy [24](#__RefHeading___Toc408902516)

REFERENCES [26](#__RefHeading___Toc408902517)

# SUMMARY

**Background:**

The largest group of visual disorders after acquired brain injury are homonymous visual field defects (HVFDs). It has been estimated that 20-30% of all patients with stroke have HVFDs [1] and that 70% of these patients show a spatially disorganized visual search strategy and disturbed visual exploration of the surroundings, impairing daily mobility-related activities [2]. As a consequence, they often fail to notice relevant objects or avoid obstacles and they are often unable or afraid to walk or cycle independently. This has far-reaching, disabling repercussions on their domestic and vocational lives and participation in society. However, when patients become aware of their deficits and learn to apply a new scanning strategy in order to compensate for the visual field defect, daily life mobility may be improved substantially [3,4]. In a recently published systematic review [3], it was concluded that compensatory scanning training is the most promising approach when it comes to the level of participation in society. This training teaches patients to apply large eye and head movements towards the blind hemifield. The patient learns to enlarge the functional field of view and to compensate for the loss of visual information that would occur if no eye movements were made.

First evidence for the effects of compensatory scanning training has been found, but the training protocols that were examined differed from each other and so far, few attention has been paid to the effects on mobility. Furthermore, only studies using within-patient repeated measures designs have been performed. The randomised controlled trial (RCT) as proposed in this study protocol provides a solution for these problems.

**Aim:** The aim of this study is to examine the effect of a compensatory scanning training. The effects will be investigated on the level of functions, activities and participation. Furthermore, the predicting effects of several variables will be examined.

**Study design:** Single-blind controlled intervention study

**Study population:** Patients with HVFD (hemianopia or quadrantanopia) due to acquired brain injury, age 18-75.

**Intervention**: All participants will receive scanning training according to a protocol at Royal Dutch Visio, Centre of Expertise for Blind and Partially Sighted People.

**Primary outcome measures:** Eye movement parameters on scanning tasks (such as fixations, saccades, fixation duration, size of saccades, etc.). See *chapter 2: Objectives and research questions* for more information.

Possible risk: The effect measures do not include invasive testing. The effect measures have no adverse consequences for the participant, nor for his/her treatment at Royal Dutch Visio and there are no risks involved. Therefore, the burden is unassuming. When patients agree to participate in the study, the only thing they need to do is come to Groningen for the effect measurements on two or three separate days. Expenses for traveling and accommodation will be compensated. Patients know they can refuse or end participation in the study at any time, without any effect on their individual treatment at Royal Dutch Visio.

Additional information:

- The study is performed by Royal Dutch Visio in collaboration with the faculty GMW of the University of Groningen. Visio is responsible for implementing the training protocol and providing training. The faculty GMW is responsible for coordinating training and effect measurements.

- The interventions will be provided at several regional departments of Visio.

- The effect measurements will take place at the department of Neuropsychology of GMW located in the University Medical Center Groningen.

# 1. INTRODUCTION

The largest group of visual disorders after acquired brain injury are homonymous visual field defects (HVFDs). It has been estimated that 20-30% of all patients with stroke have HVFDs [1] and that 70% of these patients show a spatially disorganized visual search strategy and disturbed visual exploration of the surroundings impairing daily mobility-related activities [2]. As a consequence, they often fail to notice relevant objects or avoid obstacles and they are often unable or afraid to walk or cycle independently. This has far-reaching, disabling repercussions on their domestic and vocational lives and participation in society. However, when patients become more aware of their deficits and learn to apply a new scanning strategy in order to compensate for the visual field defect, daily life mobility may improve substantially [3,4]. In a recently published systematic review [3], it was concluded that compensatory scanning training is the most promising approach when it comes to the level of participation in society. This training teaches patients to apply large eye and head movements towards the blind hemifield. The patient learns to enlarge the functional field of view and to compensate for the loss of visual information that would occur if no eye movements were made.

First evidence for the effects of compensatory scanning training has been found, but the training protocols that were examined differed from each other and so far, few attention has been paid to the effects on mobility. Furthermore, only studies using within-patient repeated measures designs have been performed. The randomised controlled trial (RCT) as proposed in this study protocol provides a solution for these problems.

First of all, a detailed training protocol will be used, that will be included in the set of available training methods at Royal Dutch Visio. During the past 10 years, Visio North has built up extensive expertise of compensatory scanning training, partly based on previous PhD research in Groningen [4]. Until now, the only department of Visio experienced with this training method was located in Haren. No standardized protocol was available for this method, causing large variety in use of the method among therapists and patients. In order to recruit enough participants for the effect study, the training will be implemented throughout The Netherlands. A protocol for the training has been written by Royal Dutch Visio Haren in order guarantee unequivocality (protocol can be requested from principal investigator G.A. de Haan). During the training, the patients’ awareness and understanding of the nature of their deficit is increased step by step and patients are taught to make eye movements towards the blind side with increasing size. After a number of sessions, these eye movements are combined with head movements and applied in mobility situations of increasing complexity. The goal is that after training, participation of the patients has increased by a decrease in mobility restrictions.

Second, multiple rehabilitation centers will participate in the RCT, making that the effects of training cannot be exclusively related to the expertise available within a certain region.

Third, the RCT as here proposed intends to demonstrate the effects of scanning training on several functional levels (cognitive and physiological) and multiple levels of participation and mobility.

Fourth, the effects of treatment will be examined by comparing a group of patients receiving treatment with a group of patients on the waiting list for the treatment. During this period of ‘waiting’, the latter group functions as a control group for the patients in the training stage. Allocation to one of the two groups will not affect the moment at which the training starts. In the medical-scientific literature, this method (RCT) is much more highly appreciated (class A studies) than within-patient repeated measures designs (class B or C studies).

**Relevance**

Guidance and training of mobility is one of the most frequently provided products in rehabilitation of people with a visual impairment. People with HVFD due to acquired brain injury often experience many problems on several levels of mobility. The need for an evidence-based training method improving mobility in people with HVFD is great.

In a recent systematic review by Bouwmeester, Heutink and Lucas [3], it was concluded that the effect of compensatory scanning training should be demonstrated in an RCT. This research proposal not only meets this scientific demand, but also aims at providing insight in the amount of transfer from trained strategies to practical mobility. The study as here proposed differs from previous studies on the effects of compensatory scanning training by using a wide variety of dependent variables, in order to evaluate the results of training on multiple levels. With eye tracking, it can be established whether compensatory scanning training actually leads to making larger eye movements. Psychophysiological and cognitive tasks can determine to what degree faster processing of and reacting to peripheral visual stimuli occurs after training. Mobility tests will be used to examine whether training leads to an improvement of practical mobility in and around the house or in slow-moving traffic. Subjective measures are used in order to determine whether training leads to an improvement in participation. Evaluation of compensatory scanning training on mobility has not been performed on this scale (using an RCT) before, let alone with these high-quality effect measures.

# 2. OBJECTIVES AND RESEARCH QUESTIONS

Primary:

The aim of this study is to examine the effect of a compensatory scanning training (CST). The effects will be investigated on the level of functions, activities and participation.

The expectation related to the training is that patients learn to make larger and more efficient saccades towards the blind hemifield, shifting potentially interesting objects into the functional healthy part of the visual field, enlarging the visual field of search and decreasing search time. Herewith we strive to improve general mobility, increasing participation and quality of life.

The following research questions have been defined:

*1. Does CST result in larger, more frequent and faster saccades to the blind hemifield?*

*2. Does CST result in more accurate and faster detection of peripheral visual stimuli?*

*3. Does CST result in shorter search times in complex visual environments?*

*4. Does CST result in safer participation in slow traffic?*

*5. Does CST result in less bumping to objects in complex visual environments?*

*6. Does CST result in a decrease in self-reported visual disabilities?*

*7. Does CST result in increased self-reported mobility?*

*8. Does CST result in improved self-reported quality of life?*

The order of presentation reflects the build-up of possible causal relations regarding the possible effect of the intervention.

Secondary:

Furthermore, the predicting effects of several variables will be examined.

The following aspects will be examined:

1. Visual functions, of which size of the visual field defect is the most important
2. Awareness and understanding the HVFD and its consequences in daily life.
3. Motivation for training
4. Presence of important health changes and life events during and preceding the study.
5. Premorbid level of intelligence
6. Executive functioning
7. Memory

# 3. DESIGN

The effects of scanning training will be examined by comparing a group of patients receiving treatment with a group of patients on the waiting list for the treatment. During this period of ‘waiting’, the latter group functions as a control group for the patients in the training stage. Allocation to one of the two groups will not affect the moment at which the training starts. In the medical-scientific literature, this method (RCT) is much more highly appreciated (class A studies) than within-patient repeated measures designs (class B or C studies).

Participants proceed through the following trajectory (randomised controlled trial design):

*chronology*

1. Admission stage at Royal Dutch Visio conform usual referral procedure via ophthalmology, rehabilitation or neurology.

2. Based on the available medical records, the project leaders and principal investigator decide on preliminary inclusion or exclusion. This is only possible in case the patient gives permission for exchanging medical information. Due to the standard procedures at Royal Dutch Visio, this is guaranteed for the large majority of cases.

3. In case of preliminary inclusion, assessments of visual and neuropsychological functioning are performed at Royal Dutch Visio. The patient receives a letter containing information about the study, as well as an informed consent form for agreeing with participation in the study in case the patient will be included.

4. The project leaders and principal investigator decide whether the patient is included or excluded based on the results of the assessments mentioned under 3.

5. In case the patient is included and has given informed consent, the patient is randomly allocated to the training group or control group (method of minimization, see for more information section *6.2 Selection*). Allocation to one of the two groups will not affect the moment at which the training starts. At Royal Dutch Visio, a waiting list of several months preceding treatment is not unusual. Patients from both the training group and the control group will visit the University Medical Center Groningen for a pre-assessment (the week before onset of training at Royal Dutch Visio) and a post-assessment (after 10 weeks of training). The control group will participate in a third assessment, 10 weeks before the pre-assessment (the so-called -10 assessment). By looking at the results from the pre-assessment and post-assessments of the training group and the results from the -10 assessment and pre-assessment of the control group, the effect of 10 weeks of training is compared to the effect of 10 weeks on the waiting list. In addition, the effect of treatment on the individual level can be examined by comparing the results from the post-assessment to the results from the -10 assessment and pre-assessment in the control group (within-patient repeated measures design). Including the post-assessment in the control group also increases power of the study.

In order to provide compensatory scanning training close to the patients’ residence, training will be provided at several locations of Royal Dutch Visio throughout the Netherlands, including Haren, Leeuwarden, Apeldoorn, Rotterdam and Haarlem. The final training protocol is composed in Haren. Therapists working at the participating departments of Visio received additional education in providing the training conform protocol. The training and diagnostic assessments take place at the department of Visio where the patient has registered.

# 4. STUDY POPULATION

## 4.1. Population

The study population consists of 60 patients of Royal Dutch Visio with HVFD (hemianopia or quadrantanopia, existing for at least 6 months) due to acquired post-chiasmatic brain injury. Participants have no severe motor, perceptual and neuropsychological disorders. Mobility (percentage preferred walking speed), participation and quality of life are important dependent variables in this study. These variables might also be influenced by other disorders than the hemianopia, such as severe balance disorders, disorders of cognitive functioning or psychological disorders (e.g. panic disorder with agoraphobia). There are several reasons to exclude patients with such disorders from the study. First of all, the comorbidity prevents us from determining the effect of the intervention. Second, in case of such comorbidity, other rehabilitation approaches are often preferred to the scanning training protocol. Because only severe comorbidity is excluded, the included group is expected to be sufficiently heterogeneous to determine the (predicting) influence of factors such as intelligence, memory and executive functioning on the training effect. Almost all patients with hemianopia that were registered at Royal Dutch Visio in the last couple of years had injury to the primary and/or secondary visual cortex, mostly because of an ischaemic accident in the acp or posterior part of the acm. Disorders of executive functioning, aphasia, spatial disorders and memory disorders are often related to damage to more frontal regions and are relatively rare in this patient group. Although hemiparesis is often present in the acute stage, patients with hemianopia rarely present motor problems when seen for rehabilitation at Royal Dutch Visio.

## 4.2. Inclusion criteria

- Homonymous visual field defects (quadrantanopia at minimum, three quarters affected at maximum, based on binocular Goldmann perimetry, isopters: V-4e, III-4e, I-4e, I-2e and I-1^e^, monocular Goldmann III-4e, and monocular Humphrey monocular 10-2), due to acquired post-chiasmatic brain injury.
- At least six months between onset of HVFD and first effect measurement
- Neurological condition is stable
- Ophthalmological condition stable for at least six months
- Age between 18 and 75 years
- Self-reported mobility-related difficulties
- Able to walk at least 50 meters independently or by using a cane/rollator, without a wheelchair (mobility assessment has to be possible, training is aimed at mobility in and around the house).
- Best corrected binocular visual acuity 0.5 (Lighthouse ETDRS 2000 chart, 4m, 500 lux, using optimal correction).
- Peak log contrast sensitivity within normal limits (Vistech VCTS6500, 4m, 500 lux, >B5 or C4)
- Eye and head mobility undisturbed in all directions
- MMSE score >25
- Sufficient memory for remembering training instructions and homework assessments (based on 8WT)

## 4.3. Exclusion criteria

- Only one functional eye
- Unclear neurological cause of HVFD
- Hemiplegia or quadraplegia
- Psychiatric disorders influencing participation, mobility and compliance with training
- Misuse of drugs/alcohol/medication
- Severe hearing impairment; hearing aids allowed, verbal communication has to be possible.
- Problems with balance or orientation impairing mobility
- Visual field defect in ‘intact’ hemi-field not connected to the main visual field defect
- Diplopia
- Metamorphosis expected to influence scanning training (Amsler test)
- Oscillopsia
- Impairments in understanding (spoken) language (based on observation)
- Severe agnosia (based on VOSP) that strongly influences the mobility problem, such as simultanagnosia
- Severe impairments of executive functioning requiring an adapted training protocol
- Severe unilateral neglect (based on Balloons, drawings, line bisection and Complex Rey Figure).
- Severe memory disorders requiring an adapted training protocol
- Strong deviance on Trailmaking, VOSP, and Balloons not explained by hemianopia
- Strong deviance on drawings and Complex Rey Figure not explained by hemianopia
- Optic ataxia
- Sticky fixation (oculomotor apraxia)

## 4.4. Sample size

The sample size was based on previous studies on the effect of CST. There is no reason to expect negative effects of training. Therefore, calculation of sample size was based on one-sided testing. Section *6.1.1 Primary outcome measures* provides more information on the reasons for eye movement parameters as primary outcome parameters. The Effect Size (ES) is calculated based on previous studies on the effect of CST on the number of fixations and refixations [2,5]. Taking the lowest value encountered (ES = 0.65), a minimum of 30 participants per group is required (training group vs. control group; two independent groups; α = 0.05; β = 0.20; one-sided testing). When comparing pre and post assessments within the total group (n=60), an ES of 0.34 can be detected with power 0.80 and one-sided testing with 0.05 significance. This means that even in case of low effectiveness of compensatory scanning training, the group size is fully sufficient. We assume an inflow of 120 patients (n=120) and we take into account a loss of 30 patients (25%) based on medical history (remaining n=90), 15 patients (17%) after assessment of inclusion and exclusion criteria (remaining n=75), and 15 patients (20%) during the period of training and effect measurements (remaining n=60).

# 5. INTERVENTION

## 5.1. Compensatory Scanning Training

The compensatory scanning training is described in a detailed treatment protocol that will be included in the set of available training methods at Royal Dutch Visio. During the past 10 years, Visio North has built up extensive expertise of compensatory scanning training, partly based on previous PhD research in Groningen. This has resulted in a detailed training protocol. During the training, the patients’ awareness and understanding of the nature of their deficit is increased step by step and patients are taught to make eye movements towards the blind side with increasing size. After a number of sessions, these eye movements are combined with head movements and applied in mobility situations of increasing complexity. The goal is that after training, participation of the patients has increased by a decrease in mobility restrictions.

# 6. METHODS

## 6.1. Study parameters

### 6.1.1. Primary outcome measures

Scanning strategy based on eye tracking (fixation and saccade parameters) on a Dot Counting Task (similar to the Dot Counting Task as used by Zihl, 1995 [2]).

The order of research questions as listed in chapter *2 Objectives and research questions*, reflects the build-up of possible causal relations regarding the possible effect of the intervention. The eye movement parameters are on the base of this build-up. After all, in case the intervention would lead to an improvement in self-reported mobility and quality of life, but not to an improvement on the eye movement parameters, it would be hard to argue that the improvement can be ascribed to specific aspects of the training. A non-specific effect of training cannot be ruled out completely in a study like this, even more so because the control group does not receive a placebo intervention. This is one of the considerations to take eye movement parameters as the primary outcome in the power calculation. A second reason was that the dependent variables in the previous studies (all within-subject repeated-measures designs) on the effect of CST mainly used these eye movement parameters on visual search tasks and not questionnaires on mobility and quality of life [3]. Although these outcome parameters are of larger clinical importance than eye movement parameters, they could not be taken as primary outcome in the power calculation, because the expected effect size could not be predicted based on the literature.

### 6.1.2. Secondary outcome measures

*Visual functions:*

- Visual field, with Goldmann (2x monocular)
- Visual acuity, with ETDRS 2000 letter chart at 500 lux
- Contrast sensitivity, with Gecko at 500 lux

*Questionnaires (see also F1.Questionnaires)*:

- Awareness; based on theories on awareness by Crosson [7] and Critchley [8] and piloted in a group of patients with hemianopia.
- Life events; questions based on the Life Events Questionnaire (LEQ) [9].
- Motivation; questions selected from Motivation for Traumatic Brain Injury Rehabilitation Questionnaire (MOT-Q) [6].
- Expectations/Evaluation of training; questions based on interviews from previous master thesis research at UMCG Beatrixoord.
- NEI-VFQ25 (National Eye Institute - Visual Functioning Questionnaire/25 items, Dutch translation)
- VOM (Vragenlijst Onafhankelijke Mobiliteit, Dutch translation of the Independent Mobility Questionnaire)
- CVD (Cerebrale Visuele Stoornissen, Dutch translation by M. L. M. Tant, 1997)

*Neuropsychological testing:*

- Grey Scales
- NLV (Nederlandse Leestest voor Volwassenen)
- Zoo-map (BADS)
- 15-Words Test

*(Ecological) scanning and mobility tests:*

- Dot Counting Task (with eye tracking); dots are presented on a large screen and are to be counted by the patients.
- Standardized search task (with eye tracking); search task on a large screen; patients have to indicate whether or not a target is present among distractors.
- Hazard perception pictures (with eye tracking); photos of traffic situations; patients have to indicate what action they would perform in the given situations: brake, release gas pedal or do nothing.
- Hazard perception movie clips (with eye tracking); short movies of traffic situations; patients have to indicate which overt and covert hazards are present.
- Tracking Task; test in which the patient tries to keep an imaginary car on the road in the presence of cross wind using a steering wheel (road is depicted on a central monitor) while simultaneously reacting on stimuli in the left and right periphery.
- Rides in a driving simulator
- Mobility test; walking through a corridor, with and without obstacles and with and without a cognitive dual task (digit span).

*Test for cross validation:*

- Reading test

Clarification: the questionnaires are used to examine the outcome on the activity and participation level: the NEI-VFQ25 (National Eye Institute - Visual Functioning Questionnaire/25 items), the VOM (Vragenlijst Onafhankelijke Mobiliteit) and the CVS (Cerebrale Visuele Stoornissen). Change in participation is also measured with the evaluation questionnaire in the post-assessment, based on evaluation questionnaires from UMCG-Beatrixoord. Finally, also the session reports from the therapists are deployed for this aim.

*Fitness to drive as outcome parameter:*

Most patients with hemianopia are not allowed to drive a car. The Netherlands are one of the few countries worldwide where patients with hemianopia can be declared fit to drive by the CBR by succeeding a special test of practical fitness to drive. In order to apply for such a driving test, the so-called ‘Eigen Verklaringsprocedure’ (www.cbr.nl) has to be followed. Every person with hemianopia has the right to apply for this procedure.

In the existent rehabilitation program ‘AutO-Mobility’, Royal Dutch Visio guides patients with visual disorders in safe and independent participation in motorized traffic. It is a close collaboration with the CBR, a number of certified driving schools, and the departments of Ophthalmology and Neuropsychology from the UMCG. Naturally, participants in this study also have the right for independent participation in motorized traffic. They may therefore not be abstained from this information. In practice, this indeed appears to be a frequent need in this population. Because training or driving lessons related to the program AutO-Mobility might interfere with the research program here proposed, both trajectories have to be carefully aligned in an early stage. This yields possibilities for examining the effect of scanning training on regaining a drivers’ license. Part of the participants in this study perform a test of practical fitness to drive at the CBR before and after scanning training. Based on these results, Visio and the CBR will decide on including the scanning training in the Eigen Verklaringsprocedure for people with hemianopia.

### 6.1.3. Other measures

The following biographical and medical variables with possible influence on the performance on the tests for social and general cognition will be included:

-Sex, age, comorbidity

-Time between acquired brain damage and first effect measurement

-Size (mm) and location of lesion based on brain imaging (CT, MRI)

These data will be derived from the medical records of the patients. For this aim, written consent will be requested from the patients.

## 6.2. Selection

The effects of scanning training will be examined by comparing a group of patients receiving treatment with a group of patients on the waiting list for the treatment. During this period of ‘waiting’, the latter group functions as a control group for the patients in the training stage.

Allocation is based on the method of minimization, under the condition that allocation of the patient minimizes the difference between both groups with regard to age, sex, side of field defect, size of field defect, time since lesion and training region.

Both groups need to be sufficiently equal regarding important prognostic variables. Differences between the groups on dependent variables should be able to be described to the intervention and not to the group characteristics. Theoretically, the allocated intervention should be the only difference between the groups. Therefore, it is highly important that potentially prognostic factors such as sex, age, lesion location and time since onset do not interfere with the interpretation of the group differences. Selection bias - an unequal distribution of these prognostic factors over the groups - forms the greatest threat to internal validity. Several different randomization techniques exist, which all serve a twofold purpose: every patient should have an equal a priori chance to receive either intervention and selection bias should be ruled out. Adaptive randomization, also called minimization, is a dynamic procedure, in which chances for allocation to one of the groups are influenced by the characteristics of the patients already allocated. The procedure is based on the idea that with every allocation, the differences between the groups are minimized [10]. The Consolidated Standards of Reporting Trials (CONSORT) state that “trials that use minimisation are considered methodologically equivalent to randomised trials, even when a random element is not incorporated” [11,12]. For the sample size of our study (n = 60), it is demonstrated that adaptive randomization is not only equivalent, but even superior to unconditional randomization [13].

The moment patients are definitely included for the study, the scanning training and all effect measurements are planned based on the waiting list at Royal Dutch Visio. Allocation to one of the two groups will not affect the moment at which the training starts. At Royal Dutch Visio, a waiting list of several months preceding treatment is not unusual.

When the waiting list is longer than 10 weeks, the patient will be allocated to either the training group or the control group based on the random allocation described above. When the waiting list is shorter, which is not expected, the patient will be allocated to the training group in case the patient belongs to this group based on the random allocation. In case the patient would belong to the control group based on the random allocation, the patient is excluded from the study, but the patient can still start scanning training within 10 weeks. Patients from both the training group and the control group will visit the University Medical Center Groningen for a pre-assessment (the week before onset of training at Royal Dutch Visio) and a post-assessment (after 10 weeks of training). The control group will participate in a third assessment, 10 weeks before the pre-assessment. (the so-called -10 assessment). By looking at the results from the pre-assessment and post-assessments of the training group and the results from the -10 assessment and pre-assessment of the control group, the effect of 10 weeks of training is compared to the effect of 10 weeks on the waiting list. In addition, the effect of treatment on the individual level can be examined by comparing the results from the post-assessment to the results from the -10 assessment and pre-assessment in the control group (within-patient repeated measures design). Including the post-assessment in the control group also increases power of the study.

The effect measurements will be performed by (student) neuropsychologists under supervision of G.A. de Haan. They are not informed about the allocation to the training group or the control group and therefore they do not know whether the patient already received training or not.

## 6.3. Study procedures

For the evaluation of inclusion, patients take part in a regular admission interview, visual function assessment, low vision assessment and neuropsychological assessment at Royal Dutch Visio in the nearest region. Participants will visit the UMCG two or three times (depending on the research group) for the effect measurements. The tests and assessments including in the effect measurements are described in section *6.1.2. Secundary outcome measures.*

## 6.4. Withdrawal of individual subjects

Patients are allowed to withdraw from the study without reason at any time, without any consequences for the individual patient. The researcher is allowed to remove patients from the study for medical reasons.

## 6.5. Replacement of individual subjects after withdrawal

In case of withdrawal, a new patient will be included.

## 6.6. Follow-up of subjects withdrawn from treatment

Patients withdrawing from the study will be asked permission for the researchers to look into the available (para)medical data.

# 7. SAFETY REPORTING

## 7.1. Section 10 WMO event

Conform section 10, sub 1, of the WMO, the researcher will inform participants as well as the accredited ethical committee (METC) about an event that suggests that participation in the study has significantly larger disadvantages than expected in the study protocol. The study will be postponed during further evaluation by the accredited ethical committee (METC), unless delay would endanger the health condition of the participants. The researcher will take care of keeping all participants informed.

## 7.2. Adverse and serious adverse events

The assessments are not expected to result in adverse or serious adverse events.

## 7.3. Follow-up of adverse events

None

# 8. STATISTICAL ANALYSIS

Alpha=0.05 will be applied for all statistical testing.

## 8.1. Descriptives

Data will be described quantitatively; averages and standard deviations. Besides quantitative scores (where possible) on the questionnaires on awareness, life events, motivation, expectations/evaluation of training and open-ended questions on the impairments due to the hemianopia, the results of these questionnaires can also be described qualitatively.

## 8.2. Univariate analysis

*Primaire onderzoeksparameter*

The effect of training on the fixation and saccade parameters on the Dot Counting Task are analyzed in two ways:

1. A between-group analysis by comparing data from T2 of the training group with data from T2 of the control group, possibly corrected for data from T1.
2. A within-group analysis by comparing change between T1 and T2 with change between T2 and T3 in the control group using repeated measures analysis.

## 8.3. Multivariate analysis

*Primary outcome measures*

Using multiple regression, a predicted model can be formulated, in which performance on the Dot Counting Task can be predicted from several variables, such as group allocation (training group vs. control group).

*Secondary outcome measures*

The effect of training on the questionnaires, scanning tests, mobility tests and the reading test will be examined using multivariate analysis (MANOVA or mixed design ANOVA), so we can control for familywise error rate.

In addition, the effect of training will be examined by comparing test performance in the control group with the preceding measurement using multivariate analysis (MANOVA for multiple repeated measures design: 1 group, 3 time points, several outcome parameters).

# 9. ETHICAL CONSIDERATIONS

## 9.1. Regulation statement

The study will be conducted according to the principles of the Declaration of Helsinki (WMA 7th revision of the Declaration of Helsinki, 59th WMA General Assembly, Seoul October 2008) and according the WMO.

## 9.2. Inclusion and consent

Patients with hemianopia will receive the first information about the study during the admission stage at Royal Dutch Visio. Depending on the region of Royal Dutch Visio, either the admission worker or the neuropsychologist will keep in touch with the patient regarding the inclusion trajectory. In case of preliminary inclusion for the study based on the available medical records, the principal investigator (G.A. de Haan) will send to the patient a letter containing information about the study, as well as an informed consent form. In case of inclusion, the patient receives additional information about the effect measurements from the principal investigator (G.A. de Haan) and information about the scanning training from the department of Planning of Royal Dutch Visio.

## 9.3. Justification of burden on participants

The effect measures do not include invasive testing. The effect measures have no adverse consequences for the participant, nor for his/her treatment at Royal Dutch Visio and there are no risks involved. Therefore, the burden is unassuming. When patients agree to participate in the study, the only thing they need to do is come to Groningen for the effect measurements on two or three separate days. Expenses for traveling and accommodation will be compensated. Patients know they can refuse or end participation in the study at any time, without any effect on their individual treatment at Royal Dutch Visio.

## 9.4. Insurance

Since no risks are involved in the research, the accredited ethical committee (METC UMCG) has provided dispensation for the insurance.

## 9.5. Compensation

Patients receive compensation for expenses for traveling and parking up to 50 euro per day of testing. Lunch will be provided.

# 10. ADMINISTRATIVE ASPECTS AND PUBLICATION

## 10.1. Handling and storage of data and documents

When included, participants receive a research number (based on chronology). This number will be connected to the personal data of the participant in a secured database (number-name-database). Only the principal investigator (G.A. de Haan) and the project leaders (W.H. Brouwer, J.H.C. Heutink and B.J.M Melis-Dankers) have access to this database. The database is saved at the RUG server and protected with a password. All data and documents collected during the study will be processed anonymously and only by consulting the number-name-database they can be connected to the person. The Informed Consent form containing name and date of birth of the participant is an exception. This form will be saved in a filing cabinet at the department of Neuropsychology. This cabinet is locked and only the principal investigator and the project leaders have access.

## 10.2. Amendments

The accredited METC will be notified about all substantial amendments in the study and has to approve these amendments before the research can be continued.

## 10.3. Annual progress report

The principal investigator will submit a summary of the progress of the trial to the accredited METC, once a year. Information will be provided on the date of inclusion of the first subject, numbers of subjects included and numbers of subjects that have completed the trial, serious adverse events, other problems, and amendments.

## 10.4. End of study report

The principal investigator will notify the accredited METC of the end of the study within a period of 8 weeks. The study has ended when the final included participant has completed the final effect measurement. In case the study is ended prematurely, the principal researcher will notify the accredited METC, including the reasons for the premature termination. Within one year after the end of the study, the researcher will submit a final study report with the results of the study, including publications and abstracts of the study to the accredited METC.

## 10.5. Publication policy

There is no formal agreement on publication of the study results by the department of Neuropsychology of the University of Groningen and the researcher (G.A. de Haan). There are no contractual obligations from the providers of grants. The intention is to publish in one or more peer-reviewed journals, as well as a dissertation.

# REFERENCES

1. Kerkhoff, G., Munssinger, U. & Meier, E. K. (1994). Neurovisual rehabilitation in cerebral blindness. *Archives of Neurology, 51,* 474-481.

2. Zihl, J. (1995). Visual scanning behaviour in patients with homonymous hemianopia. *Neuropsychologia, 33,* 287-303.

3. Bouwmeester L., Heutink, J. and Lucas, C. (2006). The effect of visual training for patients with visual field defects due to brain damage: a systematic review. *J. Neurol. Neurosurg. Psychiatry, 78*, 555-564.

4. Kooijman, A. C., Brouwer, W. H., Coeckelbergh, T. R. M., Tant, M. L. M., Cornelissen, F. W., Bredewoud, R. A. & Melis-Dankers, B. J. M. (2004). Compensatory viewing training improves practical fitness to drive of subjects with

impaired vision. *Visual Impairment Research, 6*, 1-27.

5. Pambakian, A. L., Mannan, S. K., Hodgson, T. L., Kennard, C. (2004). Saccadic

Visual search training: a treatment for patients with homonymous hemianopia.

*J.Neurol.Neurosurg.Psychiatry, 75,* 1443-1448.

6. Chervinsky, A. B., Ommaya, A. K., deJonge, M., Spector, J., Schwab, K. &

Salazar, A. M. (1998). Motivation for Traumatic Brain Injury Rehabilitation

Questionnaire (MOT-Q): Reliability, Factor Analysis, and Relationship to

MMPI-2 Variables. *Archives of Clinical Neuropsychology, 13* (5), 433–446.

7. Crosson, B., Barco, P. P., Velozo, C. A., Bolesta, M. M., Cooper, P. V., Werts, D.

& Brobeck, T. C. (1989). Awareness and compensation in postacute head

Injury rehabilitation. *J. Head Trauma Rehabil, 4* (3), 46-54.

8. Critchley, M. (1949). The problem of awareness or non-awareness of hemianopic

field defects. *Transactions of the Ophthalmological Society UK, 69,* 95-109.

9. Norbeck, J.S. (1984). Modification of recent life event questionnaires for use with

female respondents. *Research in Nursing and Health, 7*, 61-71.

10. Pocock SJ, Simon R. (1975). Sequential Treatment Assignment with Balancing

for Prognostic Factors in Controlled Clinical Trial. *Biometrics, 31,* 103-15.

11. Schulz KF, Altman DG, Moher D, for the CONSORT Group. CONSORT 2010 Statement: updated guidelines for reporting parallel group randomised trials.

12. Moher D, Hopewell S, Schulz KF, Montori V, Gøtzsche PC, Devereaux PJ, Elbourne D, Egger M, Altman DG, for the CONSORT Group. CONSORT 2010 Explanation and Elaboration: updated guidelines for reporting parallel group randomised trial.

13. Mattews, E.E., Cook, P.F., Terada, M. & Aloia, M.S. (2010). Randomizeing Research Participants: Promoting Balance and Concealment in Small Samples. *Research in Nursing & Health, 33*, 243-253.
